# Roles of tau protein in health and disease

**DOI:** 10.1007/s00401-017-1707-9

**Published:** 2017-04-06

**Authors:** Tong Guo, Wendy Noble, Diane P. Hanger

**Affiliations:** Department of Basic and Clinical Neuroscience, Institute of Psychiatry, Psychology & Neuroscience, King’s College London, London, SE5 9NU UK

**Keywords:** Tau, Microtubule binding, Alzheimer’s disease, Tauopathy, Synaptic dysfunction, Propagation

## Abstract

Tau is well established as a microtubule-associated protein in neurons. However, under pathological conditions, aberrant assembly of tau into insoluble aggregates is accompanied by synaptic dysfunction and neural cell death in a range of neurodegenerative disorders, collectively referred to as tauopathies. Recent advances in our understanding of the multiple functions and different locations of tau inside and outside neurons have revealed novel insights into its importance in a diverse range of molecular pathways including cell signalling, synaptic plasticity, and regulation of genomic stability. The present review describes the physiological and pathophysiological properties of tau and how these relate to its distribution and functions in neurons. We highlight the post-translational modifications of tau, which are pivotal in defining and modulating tau localisation and its roles in health and disease. We include discussion of other pathologically relevant changes in tau, including mutation and aggregation, and how these aspects impinge on the propensity of tau to propagate, and potentially drive neuronal loss, in diseased brain. Finally, we describe the cascade of pathological events that may be driven by tau dysfunction, including impaired axonal transport, alterations in synapse and mitochondrial function, activation of the unfolded protein response and defective protein degradation. It is important to fully understand the range of neuronal functions attributed to tau, since this will provide vital information on its involvement in the development and pathogenesis of disease. Such knowledge will enable determination of which critical molecular pathways should be targeted by potential therapeutic agents developed for the treatment of tauopathies.

## Introduction

It is estimated that more than 45 million people worldwide are living with dementia and this number is expected to increase to more than 130 million people by 2050 (http://www.alz.co.uk/research/world-report-2016). Alzheimer’s disease (AD) is by far the most common form of dementia; being more prevalent than vascular dementia, mixed dementia, Lewy body dementia (LBD) and frontotemporal dementia (FTD). In addition, other diseases clinically classified as primary motor disorders such as progressive supranuclear palsy (PSP) and Parkinson’s disease (PD), also present symptoms of cognitive decline and dementia. A key neuropathological characteristic common to these diseases is the presence in the brain of deposits of the microtubule-associated protein tau, in various morphologies, which is apparent many years before the onset of clinical symptoms [154]. To date there are no effective, disease-modifying treatments available for tauopathies, and therefore, understanding the physiological and pathological roles of tau in health and disease is important to identify new therapeutic targets. This review summarises current knowledge of the wide range of roles for tau in health and disease, extending beyond its well-known functions in microtubule binding and stabilisation.

## Tau structure and function

### The tau gene and tau isoforms

**Fig. 1:**
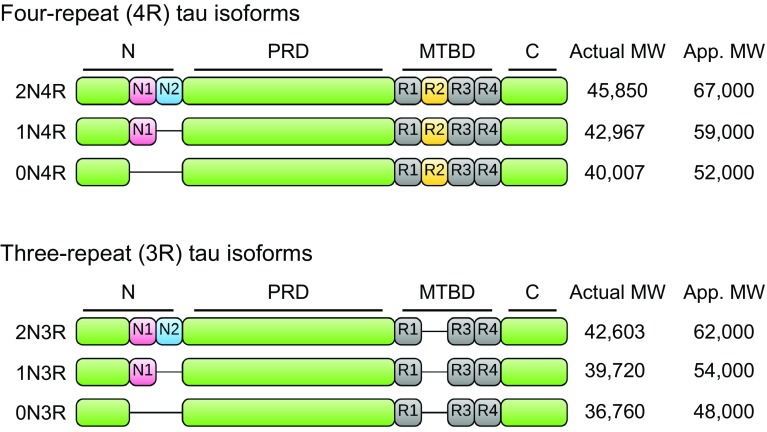
Tau protein domains and alternative splicing in the human CNS. Six isoforms of tau are generated in the human CNS by alternative splicing of the *MAPT* gene. Distinct amino acid sequences encoded by exons 2 and 3 in the N-terminal region of tau are either excluded (0N), or differentially included giving rise to 1N (exon 2) or 2N (exons 2 and 3) tau isoforms. The central region of tau comprises the proline-rich domain (PRD). Alternative splicing of exon 10 in the microtubule binding domain (MTBD), results in 3R or 4R tau isoforms. The C-terminal region is common to all six human CNS tau isoforms. The actual molecular weight (MW, kDa), and the apparent (App.) MW of each tau isoform on SDS-PAGE, are indicated on the *right*

Human tau is encoded by the *MAPT* gene, located on chromosome 17 [14]. The *MAPT* gene comprises 16 exons, although exons 0 and 14 are transcribed but not translated. *MAPT* pre-RNA is differentially spliced in a manner correlating with stages of neuronal maturation and neuronal types [511]. In the human CNS, tau protein is translated from a 6-kb mRNA transcript generating a series of six tau protein isoforms of 37–46 kDa which result from alternative splicing of exons 2, 3, and 10 (Fig. 1). These tau isoforms exhibit reduced mobility on sodium dodecyl sulphate-polyacrylamide gel electrophoresis (SDS-PAGE), such that their apparent molecular weights do not correspond to their actual molecular weights (Fig. 1). *MAPT* exons 2 and 3 each encode an insert of 29 amino acids in the amino terminal region of tau, and exon 3 is not transcribed in the absence of exon 2. Exons 4A, 6 and 8 are transcribed exclusively in the peripheral nervous system, from a 9-kb *MAPT* transcript, which is translated into a series of larger tau proteins of 110–120 kDa. Exons 9–12 encode four highly conserved imperfect repeats of 30–31 amino acids that comprise the microtubule binding domain of tau; the second repeat being encoded by exon 10. Consequently, alternative splicing yields six tau isoforms that can be differentiated by the presence of zero, one or two N-terminal inserts (0N, 1N, or 2N, respectively), and the presence of either three (3R) or four (4R) microtubule binding repeats in the C-terminal half of tau (Fig. 1).

Tau expression is developmentally regulated, such that in the adult human brain, all six isoforms of tau are expressed in the CNS, whereas in foetal brain, only the shortest tau isoform (0N3R) is expressed [164]. Approximately equal amounts of the 3R and 4R tau isoforms are present in the cerebral cortex of healthy adults [164]. Differential splicing of exons 2 and 3 results in 2N tau isoforms being relatively under-represented in comparison to 0N and 1N tau such that 0N, 1N, and 2N tau comprise 37, 54 and 9% of total human CNS tau, respectively [160]. However, the proportions of each tau isoform varies between species and in adult mouse brain, the three isoforms of 4R tau are almost exclusively expressed [260]. Furthermore, murine 3R tau isoforms are only transiently expressed in the neurons of foetal and new-born mice [306]. There are also regional differences in splicing of the *MAPT* gene in brain. For example, in humans, the amount of 0N3R tau is lower in the cerebellum than it is in other brain regions and 4R tau isoforms are increased in the globus pallidus [43, 329].

### The structural basis of tau binding to its interacting partners

The structure of tau is important for its normal functions. The amino acid sequence of the longest human CNS tau isoform (2N4R, 441 amino acids) contains a low proportion of hydrophobic amino acids relative to other proteins, rendering tau an overall hydrophilic protein [25]. The tau molecule can be subdivided into four major domains, which are distinguished by their biochemical properties (Fig. 1). The N-terminal acidic projection domain (amino acids 1–150) contains two distinct alternatively spliced N-terminal inserts. The region of tau that encompasses residues 151–243 (the proline-rich domain) [321]. The microtubule binding domain consists of four imperfectly repeated motifs, separated by flanking regions, which together provide the primary structures by which tau binds and stabilises microtubules. In contrast to the majority of the tau molecule, the second and third microtubule binding domain repeats exhibit a propensity to form an ordered β-sheet structure [354]. Finally, amino acids 370–441 form the C-terminal tail of tau.

**Fig. 2:**
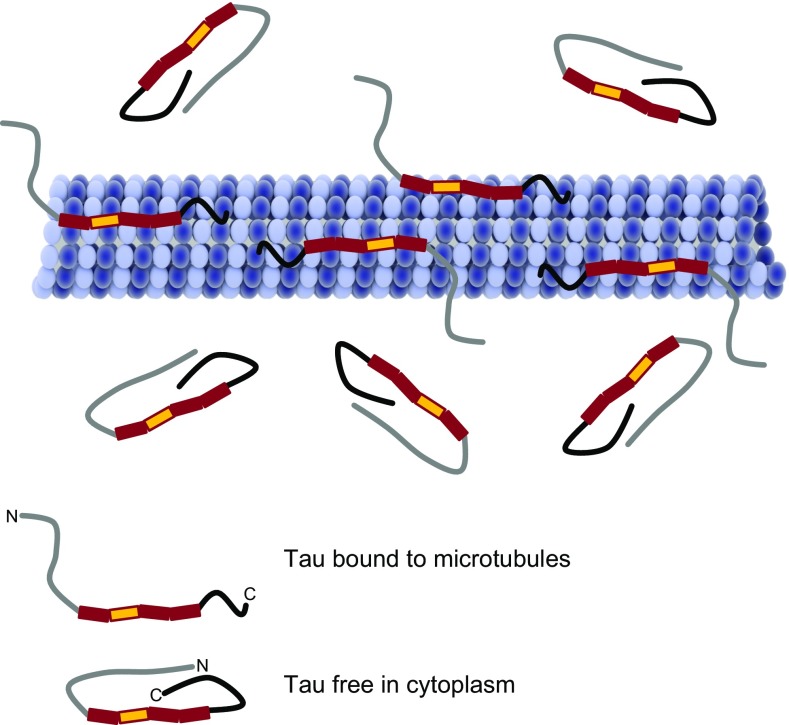
Binding of tau to microtubules. Tau associates with microtubules primarily through the microtubule binding domain, comprising either three or four repeats. The N and C termini of tau are closely associated when tau is free in the cytoplasm giving rise to the proposed “paper-clip” model of tau conformation. On binding to microtubules, the terminal regions of tau become separated and the N terminus of tau projects away from the microtubule surface

Biophysical studies have revealed tau to be a natively unfolded protein, which maintains a highly flexible conformation and overall has a low content of secondary structure [231, 354]. However, this apparent lack of well-defined secondary structure does not preclude tau folding through intramolecular interactions between its differently charged domains. Additionally, X-ray scattering, Fourier transform infrared spectroscopy, circular dichroism, and fluorescence spectroscopy also point to localised folding of tau [230]. Indeed, a “paperclip” conformation of tau has been proposed (Fig. 2), within which the C terminus folds over the microtubule binding domain and the N terminus folds back over the C terminus, bringing both termini in close proximity [230]. Notably, this association between the N terminus and the C terminus of tau is reduced upon tau binding to microtubules (Fig. 2) [408]. Moreover, tau conformation is readily disrupted by proline-directed tau phosphorylation which variably results in loosening and tightening of the paperclip structure, and this may be dependent on the specific sites of tau phosphorylation [284]. Approximately 26% of the residues in the 2N4R tau sequence are charged amino acids with a slight preponderance of positively charged residues, giving tau an overall basic character.

The N-terminal domain of tau projects away from microtubules (Fig. 2), and although this region of tau does not bind to microtubules directly, it is involved in regulating microtubule dynamics, influencing the attachment and/or spacing between microtubules and other cell components [71]. For example, N-terminally truncated tau fragments showed altered microtubule interactions, even in the presence of an intact microtubule binding domain [327]. The extreme N-terminal region of tau (residues 2–18) has been shown to be involved in a signalling cascade that inhibits axonal transport in neurons [242]. The specific functions of the N-terminal inserts in tau are not yet well established, although these sequences appear to influence the distribution of tau because 0N, 1N, and 2N tau isoforms each show distinct subcellular localisations in mouse brain [295]. Similarly, removal of the N terminus (1–150 residues) of tau promotes its localisation to the nucleus in primary rat neurons and in a human neuroblastoma cell line [381]. It has also been proposed that tau interacts with components of the neural plasma membrane through its N-terminal domain, presumably via an interaction with the membrane-binding protein annexin A2 [50, 154]. The N-terminal region of tau also binds to the C terminus of the p150 subunit of the dynactin complex, which mediates the association of the microtubule motor dynein with membranous cargoes [317]. In addition, tau isoforms in possession of different numbers of N-terminal inserts display distinct protein interaction patterns. For example, apolipoprotein A1 preferentially, if not exclusively, binds to 2N tau isoforms, whereas β-synuclein and synaptophysin more readily interact with 0N tau isoforms [296].

The proline-rich domain of tau harbours seven Pro-X-X-Pro (PXXP) motifs, providing potential recognition sites for Src homology-3 (SH3)-containing proteins including the Src family of protein kinases, such as Lck, Fgr, and Fyn, and other diverse proteins including bridging integrator 1 (Bin1), peptidylprolyl *cis*/*trans* isomerases, NIMA-interacting 1, the p85α regulatory subunit of phosphatidylinositol 3-kinase (PI3K), phospholipase C (PLC) γ1, PLCγ2, and growth factor receptor bound protein 2 (Table 1) [352]. Direct interactions between tau and SH3-containing proteins have been reported [36, 273, 278, 411, 462, 483] and these interactions are likely to have roles in modulating the signalling functions of tau. Additionally, signalling roles have been postulated from the identification of phosphatidylinositol and phosphatidylinositol bisphosphate as tau binding partners of the proline-rich domain [134, 468]. Importantly, since proline-rich regions in proteins are the target of several other protein-interacting motifs, such as WW and Enabled/VASP homology 1 (EVH1) domains, tau has significant potential to modulate signal transduction [248]. Furthermore, the proline-rich domain of tau has also been identified as a DNA and RNA interacting site [403, 507], which may be related to the identification of tau in the nucleus [54]. The proline-rich domain of tau is also involved in regulation of microtubule assembly [121, 169] and actin binding [196], indicating that this region of tau has important roles in neuronal cell signalling, nuclear function and maintenance of the neuronal cytoskeleton.

**Table 1:** Tau binding partners

Cytoskeletal	Nuclear	Signalling	Synaptic regulation	Serine/threonine-protein kinases	Tyrosine kinases	Protein phosphatases	Other tau modifying enzymes	Proteostasis	Lipid related
Actin, cytoplasmic 1	DNA	14-3-3 protein ζ/δ Zeta/delta	EF-hand domain-containing protein D2	AMP-activated protein kinase-related kinases (e.g. AMPK1, BRSK1/2, MARK1-4)	Tyrosine-protein kinase ABL1	Alkaline phosphatase, tissue-nonspecific isozyme	E3 ubiquitin-protein ligase CHIP	BAG family molecular chaperone regulator 1	Annexin A2
C-Jun amino-terminal kinase-interacting protein 1	Nucleolysin TIA-1 isoform p40	Amyloid beta A4 precursor protein-binding family B member 1	Major prion protein	Calcium/calmodulin-dependent protein kinase type IIα	Abelson tyrosine-protein kinase 2	E3 ubiquitin-protein ligase SIAH1	Histone acetyltransferase p300	E3 ubiquitin-protein ligase MARCH7	Apolipoprotein E
Dynactin subunit 1	Protein AATF	Amyloid β A4 protein	Myc box-dependent-interacting protein 1	cAMP-dependent protein kinase	Proto-oncogene tyrosine-protein kinase Src	Protein phosphatase 1	Histone deacetylase 6	Heat shock cognate 71 kDa protein	Phospholipids
Dynamin-1	Ribosomes	Calmodulin	Neuromodulin	Casein kinase 1 family (e.g. CK1δ/ε, tau-tubulin kinases 1/2)	Tyrosine-protein kinase ABL1	Protein phosphatase 2A/B	NAD-dependent protein deacetylase sirtuin-1	Heat shock protein 105 kDa	
Kinesin	RNA	Excitatory amino acid transporter 2	Synapsin-1	Casein kinase 2 family	Tyrosine-protein kinase Fgr	Serine/threonine-protein phosphatase 5	Peptidyl-prolyl *cis*–*trans* isomerase NIMA-interacting 1	Heat shock protein beta-1	
Microtubule-associated protein 1A	RNA-binding protein FUS	Growth factor receptor-bound protein 2	Synaptophysin	c-Jun N-terminal kinases	Tyrosine-protein kinase Fyn		TNF receptor-associated factor 6	Heat shock protein HSP 90-β	
Microtubule-associated protein 2	TAR DNA-binding protein 43	Phosphatidylinositol 3-kinase regulatory subunit α	α-Synuclein	Cyclin-dependent-like kinase 5	Tyrosine-protein kinase Lck		Ubiquitin thioesterase OTUB1	Hsp90 co-chaperone Cdc37	
Neurofilament light polypeptide		PLCγ1/2		Dual specificity tyrosine-phosphorylation-regulated kinase 1A				Peptidyl-prolyl *cis*–*trans* isomerase FKBP4	
Protein Hook homolog 3		Protein S100-B		Glycogen synthase kinase-3α/β				Peptidyl-prolyl *cis*–*trans* isomerase FKBP5	
Protein kinase C and casein kinase substrate in neurons protein 1				Leucine-rich repeat serine/threonine-protein kinase 2				Serine protease HTRA1	
α/β-Tubulin				Mitogen-activated protein kinases					
				Protein kinase C family					
				Serine/threonine-protein kinase Sgk1					
				Serine/threonine-protein kinases TAO1/2					

The table includes a summary of the major tau binding partners, grouped by their function or intracellular localisation. Protein names are listed in the UniprotKB database (http://www.uniprot.org/uniprot/), with the exception of some families of serine/threonine kinases and protein phosphatases [19, 323, 505]

Interactions between tau and microtubules are mediated by the microtubule binding repeats, while the flanking sequences that separate the repeats play a regulatory role in this interaction [355, 454]. Differing amino acid sequences between the four imperfect microtubule binding repeats in tau likely account for their differential affinities for microtubules [368]. Additional proteins that interact with the microtubule binding region of tau include F-actin [88], α-synuclein [233], histone deacetylase 6 (HDAC6) [114], apolipoprotein E [212], and presenilin 1 [470] (Table 1). Binding of filamentous actin occurs through a minimum of two microtubule binding repeats in tau, enabling it to link to both actin and microtubules through the repeat domain, and thereby providing an important molecular tether between the actin and microtubule cytoskeletons [123]. Such a function for tau may be important for the maintenance of healthy synapses and could therefore be critical during development, as well as in the tauopathies, particularly since this association could be disrupted by increased tau phosphorylation [143, 341]. The microtubule binding domain of tau has also been shown to associate with lipid membranes and to bind to both DNA and RNA [156, 403, 507].

Regions of tau located in both its proline-rich and microtubule binding domains are responsible for its interaction with number of neurodegenerative disease-associated proteins, including α-synuclein, 14-3-3, FUS, and TIA1 [184, 194, 488]. These findings support the view that tau is likely to have important pathological roles in disorders in which these signature proteins are deposited in the brain [194, 233].

Regarding the C-terminal region of tau, neither its function nor the proteins that bind to this domain, have been well established. However, a few studies have suggested that changes within this region might influence other domains of tau, including their interactions with other proteins and their availability for phosphorylation [86, 411].

## Post-translational modification of tau

**Fig. 3:**
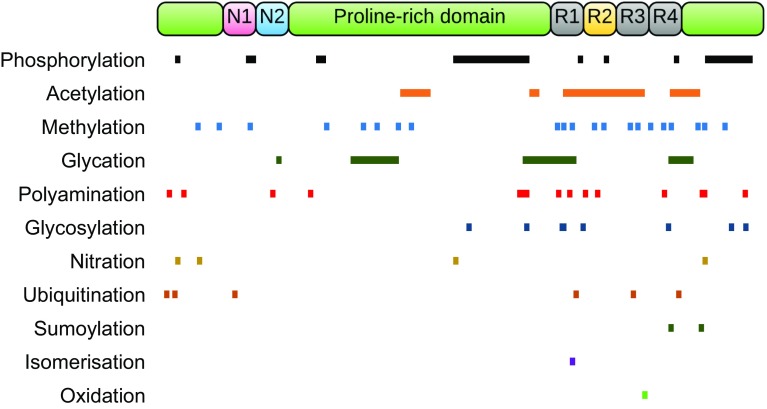
Post-translational modifications of tau. Illustration of the post-translational modifications identified on tau. The *coloured bars* indicate the approximate sites of each modification on the largest human CNS tau isoform (2N4R, 441 amino acids)

Tau is subject to a wide range of post-translational modifications, including phosphorylation, isomerisation, glycation, nitration, addition of β-linked *N*-acetylglucosamine (*O*-GlcNAcylation), acetylation, oxidation, polyamination, sumoylation, and ubiquitylation (reviewed in [323, 351]) (Fig. 3). Hence, many different tau binding partners share the property of being regulatory components of post-translational modification, such as protein kinases and phosphatases. Tau is also a substrate for the ubiquitin–proteasome system (UPS) and for chaperone-mediated autophagy [416].

### Tau phosphorylation

Phosphorylation is the most commonly described post-translational tau modification. Tau contains 85 putative phosphorylation sites, including 45 serine, 35 threonine, and five tyrosine residues, which comprise 53, 41, and 6% of the phosphorylatable residues on tau, respectively [188]. Given the large number of potential phosphorylation sites on tau, it is not surprising that phosphorylation has a profound impact on its physiological function. Under pathological conditions, tau phosphorylation is increased, which reduces its affinity for microtubules, resulting in cytoskeleton destabilisation, particularly in neurons. Tau phosphorylation at Ser262, Ser293, Ser324 and Ser356, located in equivalent positions in each of the four microtubule binding repeats, decreases tau binding to microtubules [118]. In vitro studies have shown that phosphorylation at Thr214, Thr231 and Ser235 also contributes to the dissociation of tau from microtubules [266, 442]. These findings indicate that regions of tau lying outside the microtubule binding domain also influence the association of tau with the cytoskeleton.

There is a long-established link between abnormal phosphorylation and self-aggregation of tau. Tau phosphorylation decreases tau binding to microtubules and reduces microtubule stability. The detached tau then undergoes self-aggregation, forming oligomers and higher order tau aggregates [239, 498]. It is not yet known which of the many tau phosphorylation sites that have been identified are essential for disease pathogenesis and which sites may become phosphorylated only after the formation of tau pathology in the tauopathies. Mimicking permanent tau phosphorylation by substituting phosphorylatable residues with negatively charged glutamate or aspartate (pseudophosphorylation or phosphomimicking), reproduces some of the structural and functional aspects of the pathologically phosphorylated tau observed in AD brain and exerts neurotoxic effects, including caspase activation and initiation of apoptosis [167]. Tau phosphorylation in the proline-rich region disrupts its microtubule assembly activity and induces a subtle increase in the propensity of tau to self-aggregate [121]. In contrast, phosphorylation of the C-terminal region of tau markedly promotes tau self-aggregation [300]. These reports suggest that site-specific tau phosphorylation serves to differentially regulate both microtubule binding and tau aggregation.

Several lines of evidence indicate that increased tau phosphorylation might induce neurodegeneration through mechanisms other than loss of microtubule binding function or gain of toxic oligomeric or aggregated tau species. First, elevated tau phosphorylation detaches tau from microtubules and also induces tau missorting from axons into the somatodendritic compartment, compromising axonal microtubule integrity and inducing synaptic dysfunction [205, 226]. Second, phosphorylation of tau can disrupt its intracellular route of degradation. For example, tau phosphorylated on Ser262 or Ser356 is not recognised by the C terminus of heat shock protein 70-interacting protein-heat shock protein 90 (CHIP-HSP90) complex and is thereby protected from degradation by the proteasome [111]. In contrast, phosphomimic tau is selectively cleared by autophagy compared to endogenous tau [416]. Third, microinjection of tau into synaptic terminals increases calcium and disrupts synaptic transmission through a mechanism involving kinase activation [347]. Finally, phosphorylation alters the association of tau with its interacting partners, such as cytoplasmic membrane, DNA and Fyn, disturbing the functions of tau in a range of signalling pathways [188]. However, recent evidence has suggested a protective role for tau phosphorylation against Aβ-induced toxicity [224]. In an AD mouse model generated based in APP23 mice, which expresses APP with both the Swedish and London (V717I) mutations, specific tau phosphorylation at Thr205 disrupted the assembly of PSD-95/tau/Fyn complexes, a complex required to mediate Aβ toxicity [224, 226].

#### Tau kinases

Tau phosphorylation is tightly controlled by the balance between protein kinases and phosphatases [188]. Tau kinases can be classed into three broad groups: (1) proline-directed serine/threonine-protein kinases, including glycogen synthase kinase (GSK) 3α/β, cyclin-dependent kinase-5 (Cdk5), mitogen-activated protein kinases (MAPKs), and several other kinases including those activated by stress; (2) non-proline-directed serine/threonine-protein kinases, such as tau-tubulin kinase 1/2 (TTBK1/2), casein kinase 1 (CK1), dual-specificity tyrosine phosphorylation regulated kinase 1A (DYRK1A), microtubule affinity-regulating kinases (MARKs), Akt/protein kinase B, cAMP-dependent protein kinase A (PKA), protein kinase C, protein kinase N, 5′ adenosine monophosphate-activated protein kinase (AMPK), calcium/calmodulin-dependent protein kinase II (CaMKII), and thousand and one amino acid protein kinases (TAOKs) 1 and 2, and (3) protein kinases specific for tyrosine residues, such as Src, Fyn, Abl, and Syk [323].

More than 40 putative phosphorylation sites in tau have been identified as targets of GSK3, with at least 29 of these residues being phosphorylated in AD brain [188]. Both the total protein amount and the activity of GSK3 in tauopathy brain appears to correlate with the progression of neurodegeneration, and over-activation of GSK3β significantly contributes to tau phosphorylation [386]. Moreover, GSK3 activity correlates with neurofibrillary tangle burden in AD [284] and GSK3β colocalises with neurofibrillary pathology in AD brain [191]. Tau phosphorylation by GSK3β has also been shown to induce tau aggregation [406]. GSK3β phosphorylates tau at Thr231 and primes residues in the C terminus of tau for subsequent phosphorylation, thereby providing a potential mechanism through which pathological tau phosphorylation and aggregation occurs [75]. In transgenic mice, inhibiting GSK3β reduces tau phosphorylation, tau pathology development, axonal degeneration [57, 283, 370], and rescues neuronal loss [444]. Taken together, these data suggest that inhibition of GSK3β could be a promising therapeutic strategy for AD. However, clinical trials of GSK3 inhibitors have not shown positive results and it is unclear whether targeting specific mediators of tau phosphorylation will provide an effective therapy for the tauopathies [370].

In addition to GSK3, other kinases such as Cdk5, p38MAPK, CK1δ, PKA, DYRK1A, and TAOKs may be involved in tangle formation in the tauopathies. For example, an association between Cdk5, tau phosphorylation and neurofibrillary degeneration has been established in transgenic mice with aberrant Cdk5 activity [95, 371]. Several MAPKs phosphorylate tau and some colocalise with tangles in AD brain [541]. CK1δ may also be an important candidate tau kinase since it phosphorylates tau on 46 sites [189] and colocalises with tau pathology in AD brain [440]. DYRK1A phosphorylates tau on three sites and inhibiting DYRK1A has recently been proposed as a therapeutic approach for AD [91]. Notably, the ability of DYRK1A to phosphorylate Thr212 on tau, implicates DYRK1A as a potential priming kinase, facilitating subsequent GSK3β phosphorylation of tau on the nearby residue Ser208 [426]. Similar to GSK3, TAOKs 1 and 2 each phosphorylate tau on more than 40 residues, and have many overlapping sites [474]. Also, activated TAOKs colocalise with tangles, suggesting a potential role for these kinases in the development of tau pathology in AD brain [474].

Tau is phosphorylated on five tyrosine residues at Tyr18, Tyr29, Tyr197, Tyr310, and Tyr394 [110, 274, 436]. A number of these tyrosine residues are also phosphorylated by Src family kinases, such as Src, Lck, Syk, Fyn and c-Abl [110, 411]. Phosphorylation of Tyr18, a site targeted by Fyn kinase, has been proposed to regulate axonal transport [93, 241]. The tyrosine phosphorylation state of tau also appears to correlate with its propensity to aggregate [188, 490]. Tyrosine phosphorylation of tau at Tyr18 has also been detected in soluble and detergent-insoluble preparations of FTD brain and in spinal cord from mice expressing human tau with the P301L mutation, which is one of the many tau mutations responsible for the development of frontotemporal lobar degeneration characterised by tau-positive inclusions (FTLD-tau) [490]. Interestingly, Tyr18 phosphorylation appears to have diverse effects in different neurodegenerative conditions. Tyr18 phosphorylation of tau occurs concurrently with an increase in phosphorylation at the AT8 epitope, an established marker of tau pathology, in transgenic mice expressing P301L tau, but not in 3xTg-AD mice, or in AD brain, in which β-amyloid (Aβ) deposition occurs alongside tau pathology [35, 277]. These findings imply that the role of tau tyrosine phosphorylation might vary between different diseases. In addition, phosphorylation of tau at Tyr18 is required for Aβ-induced cell cycle re-entry, another pathological effect that could be involved in the process leading to neuronal cell death [446]. A key role for the interaction of tau with tyrosine kinases was demonstrated in mice overexpressing amyloid precursor protein (APP), which exhibit a significantly increased Aβ load, and in which tau was shown to mediate Aβ-induced excitotoxicity through its interaction with Fyn tyrosine kinase [226].

#### Tau phosphatases

Protein phosphatase 2A (PP2A) accounts for more than 70% of cellular phosphatase activity in the brain [297]. PP2A dephosphorylates tau and is implicated in the regulation of tau phosphorylation [168]. PP2A activity is decreased by approximately 50% in AD brain, which could contribute to increased tau phosphorylation [297]. Incubation of misfolded tau isolated from AD brains with PP2A restores tau binding to microtubules to a level similar to that of recombinant 2N4R tau [504]. Another protein phosphatase, PP5, also dephosphorylates tau and its activity is reduced by 20% in AD brain [299]. It is worth mentioning that, in some cases, PP2A activity can override the kinase activities of GSK3β and Cdk5 with respect to tau [394]. These findings indicate that both down-regulation of tau dephosphorylation and excess phosphorylation of tau are implicated in the aberrant phosphorylation of tau observed in tauopathy brain.

PP2A has been reported to dephosphorylate GSK3β at Ser9 [281], and conversely, activation of GSK3β can inhibit PP2A [529]. Importantly, Akt inhibits GSK3β and hence plays a critical role in maintaining the balance between the activities of GSK3β and PP2A [94]. Thus, attenuation of PI3 K/Akt signalling, such as has been reported in AD brain, increases GSK3β activity and elevates tau phosphorylation and tangle formation. The mammalian target of rapamycin (mTOR) also regulates the activity of PP2A, since inhibiting mTOR results in PP2A activation [334]. These findings suggest the existence of a regulatory loop involving PP2A, mTOR, Akt, and GSK3β, which serves to maintain the phosphorylation status of tau. Hence, when Akt/mTOR signalling is adversely affected, this signalling pathway may also become perturbed, with consequences for tau phosphorylation and function [334].

### Tau acetylation

Acetylation of tau is emerging as an important post-translational modification relevant to both its physiological and pathological functions [511]. Tau acetylation is mediated by cAMP-response element binding protein (CREB)-binding protein (CBP) [340], whereas sirtuin 1 (SIRT1) and HDAC6 are responsible for tau deacetylation [87]. Notably, tau also has an intrinsic acetyltransferase activity, catalysing auto-acetylation mediated by cysteine residues 291 and 322, located within the second (R2) and third (R3) microtubule binding repeats of tau, respectively (Fig. 1) [83]. Studies examining the isolated microtubule binding domains of tau have suggested that this auto-acetylation is dependent on the close proximity of the target lysines located at residues 274 and 340 [315]. Furthermore, auto-acetylation of tau facilitates the fragmentation of tau and possibly enhances its autophagic degradation [82]. CBP acetylates tau at several lysine residues within the microtubule binding repeats and the proline-rich region, whereas auto-acetylation occurs preferentially at lysine residues located in the microtubule binding repeats [82]. Acetylation of tau lysine residues 259, 290, 321 and 353 occurs in control human brain, and appears to both protect tau from increased phosphorylation and suppress tau aggregation [87]. Conversely, acetylation of these lysine residues is reduced in AD brain and that of rTg4510 transgenic mice, that regulatably over-express FTLD-causing, P301L tau [87]. Acetylation of tau at lysines 174, 274 and 280 has been detected in post-mortem AD, Pick’s disease (PiD), FTLD-tau, and PSP brain, and acetylation at these sites may be pathological [221, 339]. This view is supported by the observation that acetylation of lysines 163, 280, 281 and 369 inhibits proteasome-mediated tau degradation, leading to the accumulation of highly phosphorylated tau [84, 340, 351]. Acetylation of tau at Lys280 in particular appears to retard tau turnover, which may be critical for tau-induced toxicity [339, 481]. Furthermore, aberrant acetylation of tau at Lys274 and Lys280 has been detected in brain tissue from rTg4510 mice [461, 481]. Interestingly, acetylation of tau at Lys274 has been widely observed across the majority of human tauopathies, with the exception of the 4R tauopathy, argyrophilic grain disease (AGD) [178].

Mutant constructs that either mimic or block tau acetylation by substitution of specific Lys residues with Gln or Arg, respectively, have provided powerful tools with which to examine the role of tau acetylation in neurodegeneration. In a *Drosophila* transgenic model, mimicking tau acetylation at Lys280 exacerbated neurotoxicity caused by tau overexpression, and altered tau phosphorylation, resulting in locomotor defects and photoreceptor neurodegeneration without altering tau solubility [171]. Importantly, tau acetylation also impacts upon synaptic function. Mice expressing pseudo-acetylated (lysine substituted with glutamine) human tau at Lys274 and Lys281 (K274/281Q) display memory deficits and impaired hippocampal long-term potentiation (LTP) [481]. Such synaptic dysfunction has been attributed to reduced amounts of the memory-related protein KIdney/BRAin (KIBRA) in transgenic mouse and AD brain [481], as well as to disruptions in activity-induced post-synaptic actin remodelling and α-amino-3-hydroxy-5-methyl-4-isoxazolepropionic acid (AMPA) receptor membrane insertion [197]. Tau acetylation is also associated with destabilisation of the axon initial segment (AIS), which separates the soma and dendrites from the axon in neurons [458]. In primary neuronal cultures, expression of the tau acetylation mimic, K274/281Q, compromised the cytoskeletal network in the AIS, leading to the missorting of axonal K274/281Q tau into the somatodendritic compartment. In AD brain, reduced ankyrin and β-spectrin, which are components of the AIS, correlates with increased tau acetylation at Lys274 and Lys281 [458]. These findings suggest that pathological increases in acetylated tau destabilise the cytoskeletal network, resulting in tau mislocalisation in the somatodendritic compartment. Accumulation of acetylated tau in dendrites could disrupt the expression of KIBRA and AMPA receptor membrane insertion, leading to synaptic dysfunction and ultimately cognitive impairment.

These findings raise important questions about the means by which pathological tau acetylation is triggered, and how this modification impacts on synaptic function and the development of tau pathology in human disease. It is intriguing to speculate that the impact of tau acetylation on its function may be either beneficial or detrimental depending on the target residue and on the relative contributions of enzyme-mediated acetylation and auto-acetylation of tau. Since tau acetylation markedly influences the capacity of tau to become phosphorylated and aggregated, developing strategies to correct tau acetylation could represent a new therapeutic approach for the treatment of human tauopathy.

### Other tau modifications

In human AD brain, but not in control brain, tau is modified by N-glycosylation, which is proposed to be involved in maintenance of the structure of neurofibrillary tangles [503]. Furthermore, N-glycosylation may facilitate tau phosphorylation, by suppressing dephosphorylation to accelerate tau phosphorylation, most likely by affecting tau conformation [301]. The mechanisms leading to N-glycosylation of tau in AD is unclear, however, it is feasible that alterations in the localisation of tau could result in aberrant glycosylation, which could affect tau function by increasing its phosphorylation.

In contrast, addition of *O*-linked *N*-acetylglucosamine (*O*-GlcNAc), which occurs on serine and threonine residues in tau, may protect it from phosphorylation, since this modification has been proposed to compete with tau kinases to modify the same target amino acids [298, 457]. In addition, *O*-GlcNAcylation can suppress tau aggregation [531], and hence, the reduction in tau *O*-GlcNAcylation observed in AD brain might contribute to the increased phosphorylation and aggregation of tau [298]. Recently, it has been shown that *O*-GlcNAc transferase, the enzyme responsible for *O*-GlcNAcylation, is significantly reduced in AD brain [502]. Moreover, mice in which expression of *O*-GlcNAc transferase was knocked out in forebrain exhibit cognitive impairment, along with neurodegeneration and increased tau phosphorylation [502], suggesting that targeting of *O*-GlcNAcylation might represent an effective therapeutic strategy for tauopathy.

Other types of post-translational modifications, including glycation, deamidation and isomerisation, have also been detected in tau extracted from AD but not from control brain [515]. All of these modifications may facilitate tau aggregation, potentially by altering tau conformation [275, 515]. Furthermore, glycation of tau may reduce the binding of tau to microtubules [410].

Abnormal nitration of Tyr18, Tyr29 and Tyr394 in tau has been detected only in AD and other tauopathies. Nitration of these residues alters the conformation of tau, reducing its ability to bind to microtubules, and depending on the nitration sites can either promote or inhibit tau aggregation [410].

Notably, tau is ubiquitylated through Lys48 linkages by the action of CHIP or tumour necrosis factor receptor-associated factor 6 (TRAF6), leading to proteasomal degradation of tau [392]. Increased tau ubiquitination also occurs in tauopathies. Interestingly, a competition between acetylation and ubiquitination of specific lysines in tau has been suggested in neurons, HEK293 cells and also in wild-type mice [340, 351]. Notably, 11 of the 14 acetylation sites identified in wild-type mice are also sites of ubiquitination in tau, suggesting that ubiquitination-dependent tau degradation could be directly affected by tau acetylation [351]. Tau is also a substrate for sumoylation, with Lys340 being the major target site [313]. Sumoylation of tau by small ubiquitin-like modifier protein 1 (SUMO-1) counteracts the effects of ubiquitylation and correlates with increased tau phosphorylation, at least in cultured cells [313]. Moreover, in AD brain, SUMO-1 colocalises with phosphorylated tau [313]. Hence, it is likely that sumoylation promotes tau phosphorylation and inhibits ubiquitin-mediated tau degradation, which could also contribute to the development of tau pathology in the tauopathies.

Finally, methylation of tau on both lysine and arginine residues has recently been described [144]. Although the functional implications of tau methylation have not been established, tau methylation occurs on many of the same lysine residues as does acetylation and ubiquitination [527]. It is conceivable that lysine methylation within the Lys-X-Gly-Ser (KXGS) motifs in the microtubule binding domain could reduce the ability of tau to bind and stabilise microtubules, and potentially also modulate tau aggregation. In addition, some lysine sites are both mono-methylation and di-methylation recognition sites and the specific modification would result in recognition by different methyl-binding domain proteins.

Collectively, there are at least four potentially competing modifications of tau that occur on lysine residues (glycation, acetylation, ubiquitination, and methylation), which highlights the strategic role of lysine modification in tau function.

In summary, there are a wide variety of post-translational modifications that can be present on tau in both physiological and pathological states, as well as many different sites that can be affected by these alterations. This combination of factors makes it difficult to identify the most important pathways that modify tau and how these might be differentially affected in health and disease.

## Tau localisation in neurons

**Fig. 4:**
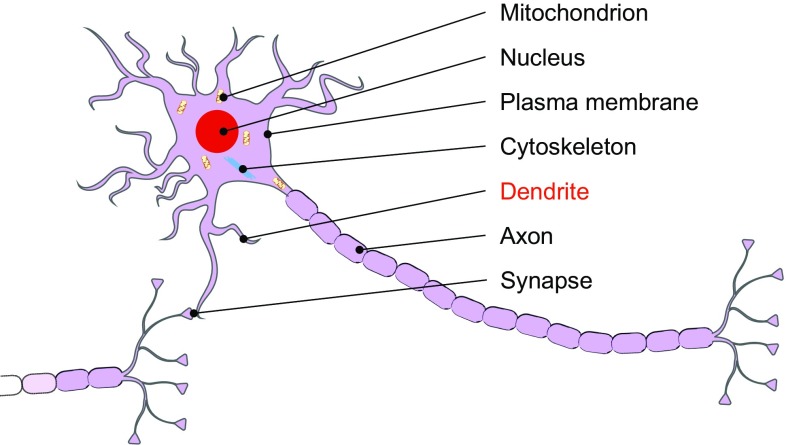
Tau localisation in neurons. Schematic depicts the differing locations of neuronal tau, the majority of which is associated with the microtubule cytoskeleton in axons. Tau is also located in the somatodendritic compartment, including in mitochondria, the nucleus, plasma membrane, and synapses. Dendritic tau (indicated in *red*) is increased in the tauopathies

Under physiological conditions, tau in human brain is expressed in neurons and to a lesser extent in oligodendrocytes and astrocytes [356, 383]. Intraneuronal tau is mainly located in axons [352] and in much lower amounts in somatodendritic compartments [473], including the plasma membrane, nucleus and mitochondria [290] (Fig. 4). Several possible mechanisms have been proposed to contribute to the polarised distribution of tau within neurons. First, tau mRNA is specifically targeted to the axonal compartment by the axonal localisation signal sited within the 3′-untranslated region of the *MAPT* gene [21]. Following the transport of *MAPT* mRNA into the axon, tau translation can be specifically upregulated, due to the presence of a 5′-terminal oligopyrimidine tract which is recognised by the mechanistic target of rapamycin-p70S6 kinase (mTOR-p70S6 K) pathway [349]. In addition, cytosolic tau can translocate to axons, either through free diffusion between the cytosol of different compartments or by motor protein-driven tau transport [258]. Tau molecules can also diffuse along microtubules guided by the microtubule lattice [201]. Alternatively, tau can be actively transported by motor proteins such as kinesin family members [484, 485]. Retention of tau in the axon is ensured by (1) maintaining a relatively low level of tau phosphorylation in axons, which increases its binding to axonal microtubules and (2) a functional axon initial segment, which forms a retrograde barrier, allowing tau to enter the axon but preventing it from travelling back towards the soma and dendrites [289].

### Cytoskeletal localisation of tau

In adult neurons, tau is mainly distributed in axons, where it interacts with microtubules. Upon binding, tau stabilises microtubules either directly, or through acting as a cross bridge which enables microtubules to interconnect with other cytoskeletal components such as actin and neurofilaments [1].

Tau can also serve as a direct inhibitor of HDAC6, which deacetylates tubulin, and inhibiting HDAC6 may thereby enhance microtubule stability [388]. However, reports are discordant on the amount of acetylated tubulin present in tau knockout mice, with some suggesting that tubulin acetylation is increased following tau deletion and others reporting no change in acetylated tubulin between tau knockout mice and wild-type controls [388, 408]. Thus, tau can influence microtubule stability by mechanisms that are both dependent and independent of its ability to bind to tubulin.

### Dendritic and synaptic localisation of tau

Under physiological conditions, tau is located mainly in axons [21] and in significantly lower amounts in dendrites, including dendritic spines [226, 255]. The physiological role of tau in dendrites is not well understood, however, a recent study has implicated tau in regulating synaptic plasticity in hippocampal neurons in response to brain-derived neurotrophic factor [73]. Furthermore, tau translocation to the post-synaptic compartment is dependent on neuronal activity [136]. Importantly, a novel role for tau in the morphological and synaptic maturation of new-born hippocampal granule neurons has recently been reported [382]. Tau is required for the proper formation of post-synaptic densities, dendritic spines, and mossy fibre terminals and knocking out tau also reduces the sensitivity of new-born granule neurons to modulators of neurogenesis [382]. Notably, a recent study has shown that tau is involved in regulating the somatodendritic localisation and protein interactome of TIA1, an RNA-binding protein [488]. Tau is also involved in the formation, size and trafficking of stress granules, which has important implications both for the neuronal response to stress and for the pathogenesis of several neurodegenerative diseases [488]. Current evidence suggests that both the formation and trafficking of stress granules are modulated by tau which reduces the rate at which stress granules are trafficked in neurons [488]. However, since stress granules are transported on microtubules, the possibility cannot be excluded that defective trafficking could be caused by impaired tau-mediated stabilisation of microtubules in disease.

### Association of tau with neuronal membranes

The N-terminal projection domain of tau is involved in regulating its interaction with the plasma membrane, in a process mediated by annexin A2 [50, 154]. However, a recent structural analysis has identified specific regions located in the microtubule binding domain of tau that bind to lipid bilayers, indicating that multiple domains of tau might associate with membranes [156]. Tau has also been shown to be recruited to membranes by Fyn kinase, localised in lipid rafts [256]. The functional relevance of the association of tau with membranes is not well established but a role in neurite development, presumably by bridging the growing microtubules to the membrane cortex in the growth cone, has been suggested [154]. This view is supported by the observation that expression of a tau mutant capable of binding to Fyn, but lacking the microtubule binding domain, reduced both the number and the length of the processes elaborated by oligodendroglia [256]. Interactions between tau and membranes are also required for targeting tau to the cell surface to enable tau to participate in intracellular signalling pathways [397]. At the cell surface, tau can interact with proteins involved in synaptic signalling, such as GluR2/3 subunits of the AMPA receptor [254].

Importantly, the association of tau with the plasma membrane is regulated by tau phosphorylation state [399, 483]. Plasma membrane-associated tau is present in a relatively dephosphorylated state in SH-SY5Y neuroblastoma cells, PC12 cells exogenously expressing tau, and cortical neurons [122, 399]. Furthermore, phosphorylation of tau, either directly or using pseudo-phosphorylated tau mutants in cultured cells, abolishes its interaction with cell membranes [316, 399]. Such effects may be caused either by conformational changes effected by tau phosphorylation, or by altered interactions with other membrane-binding proteins, such as Fyn tyrosine kinase [229, 411].

Interestingly, both in vivo and in vitro evidence has shown that tau-membrane interactions appear to correlate with tau aggregation [237]. One possible explanation for this finding is that direct binding of the microtubule binding domain of tau to the lipid surface of the membrane appears to alter tau secondary structure, which facilitates its aggregation [156]. Displacement of tau from microtubules, caused by increased tau phosphorylation or increased association of tau with phosphatidylserine in neuronal membranes, could result in increased tau aggregation [450]. However, direct evidence obtained from cell or animal models of tauopathy is still needed to confirm this hypothesis, and to establish the precise role of membrane-associated tau in neurons.

### Nuclear tau

Nuclear tau has been reported in a wide variety of cell and animal systems, including in control and AD brain, human and rat neuroblastoma cells, and human non-neuronal cell lines [54]. To date, the transcript encoding nuclear tau has not been conclusively identified. There is evidence that the majority of nuclear tau may have comprised a specific isoform, possibly encoded by a transcript distinct from the 6 kb species, which encodes the six tau isoforms in the human CNS [509]. In support of this view, in murine brain the 1N4R tau isoform preferentially localises to the nucleus, with some also present in the soma and dendrites, but not in axons [295]. Other tau isoforms are also present, albeit in low amounts, within the nucleus [295]. Interestingly, phosphorylation impacts on the behaviour of nuclear tau, especially its intranuclear localisation [54]. Reports indicate the existence of both phosphorylated and non-phosphorylated tau in the nucleus [49, 177, 451], although it appears that the majority of nuclear tau present is in a non-phosphorylated form [309, 509].

In vitro studies have shown that tau can bind DNA and thereby increase its melting temperature [60]. Similar to its ability to bind microtubules, tau binding to DNA is dramatically reduced upon tau phosphorylation [403]. Tau binds to double stranded DNA in cooperation with histones, and shows little or no sequence specificity, whereas binding of tau to single stranded DNA is sequence-specific [211, 265]. Binding to DNA is thought to be associated with the ability of tau to protect against hydroxyl free radical-induced DNA breakage [60, 312]. In support of this notion, tau in primary cortical neurons displays several characteristics reminiscent of heat shock protein 70 (HSP70) [467]. On exposure to heat stress, cytoplasmic tau translocates to the nucleus, where it protects the integrity of DNA. In contrast, knocking out tau renders cortical neurons vulnerable to heat stress-induced DNA damage, and this vulnerability is mitigated by overexpression of tau [467]. Similarly, tau knockout neurons are more susceptible to hyperthermia-induced DNA and RNA breakage in comparison to their wild-type counterparts [495]. Furthermore, tau may be involved not only in DNA protection, but also in DNA repair mechanisms [495], although this remains controversial since others have reported that tau is not involved in DNA repair [421].

In addition to its function of protecting DNA, tau also showed potential as a modulator of gene expression. Tau binds to the AT-rich minor groove of DNA through its proline-rich and microtubule binding domains [403, 421].The typical function of minor groove architectural binding proteins, such as high mobility group proteins, is to alter DNA conformation, causing it to unwind [34]. This altered DNA conformation enhances the assembly, stability and activity of multi-protein-DNA complexes, and indirectly either enhances or inhibits gene transcription [34]. Hence, it is possible that the interaction of tau with DNA could initiate the formation of a multi-protein complex in a similar fashion to that of other minor groove binding proteins [177]. Indeed, the capacity of tau to change the conformation of DNA has been reported [467], resulting in modulation of gene expression. Genetic analysis of tau knockout mice suggests that tau could have an indirect effect on gene transcription, likely through compensatory changes in gene expression. To date, the transcription of at least 14 genes have been reported to be significantly increased following tau depletion, all of which have been verified by microarray analysis in combination with quantitative real-time PCR [101, 379].

In addition to affecting DNA conformation and thereby gene transcription, tau colocalisation with histones provides potential links between tau and organisation of heterochromatin, as has been observed in human skin fibroblasts and HeLa cells as well as in tau transgenic *Drosophila* and mice, and in AD [139, 456]. Histones and tau protein both bind to the minor groove of DNA and show similar effects in DNA retardation assays [60]. A recent study has revealed that tau binds to and localises either within or adjacent to neuronal heterochromatin in primary neuronal cultures from wild-type mice [322]. In tau knockout mice, the distribution pattern of the trimethylated forms of histone H3 and heterochromatin protein 1α are disrupted. These findings support the view that tau may have a role as an epigenetic regulator of gene expression. In addition, tau is reported to contribute to chromosomal stability and to participate in the processing and/or silencing of ribosomal RNA [309, 421].

In summary, it is becoming increasingly evident that nuclear tau plays roles in DNA protection, preserving its integrity, and possibly participating in DNA repair mechanisms. In addition, tau in the nucleus can regulate genomic function. However, tau is also reported to participate in DNA damage responses, thereby dysregulating transcription [495]. Further research is needed to resolve these potentially discrepant findings.

## Tau and neuronal activity

Pathological changes observed following depletion of murine tau have indicated the involvement of tau in the regulation of neuronal activity, neurogenesis, and long-term depression [255]. Tau knockout mice exhibit a selective deficit in long-term depression, although not in long-term potentiation (LTP), in the Cornu Ammonis 1 (CA1) region of the hippocampus, indicating a role for tau in synaptic plasticity [255]. Removal of tau leads to decreased migration of new-born neurons from the subgranular zone of the hippocampal formation to the granular layer, suggesting a role for tau in neuronal migration [145]. Moreover, abolishing tau expression in adult mice results in a severely impaired hippocampal neurogenesis [204], which may be related to the requirement for a dynamic microtubule cytoskeleton for efficient neurogenesis [146]. Importantly, recent investigation of neurogenesis in tau knockout mice has elucidated new roles for tau in regulating the functional maturation and survival of new-born neurons, the selectivity of neuronal death following stress, and neuronal responses to external stimuli [382].

However, it is notable that the phenotypic changes exhibited by different lines of tau-deficient mice have proved to be somewhat inconsistent due to several possible confounding factors (reviewed in [27]). First, changes induced by the absence of tau during neuronal development may be variably compensated by increased expression of other microtubule-associated proteins, including MAP1A. Second, one tau knockout mouse line expresses part of tau exon 1, which could interact with tau binding proteins and/or membrane components. Third, some motor abnormalities observed in mice lacking tau appear to be age-related, and possibly associated with effects on the peripheral nervous system in some lines. Finally, phenotypic variation of different mouse lines can be strongly influenced by the specific mouse background used. Therefore, although tau-deficient mice are valuable models for assigning novel functions of tau, such findings need to be validated in multiple lines of mice.

## Tauopathies

**Fig. 5:**
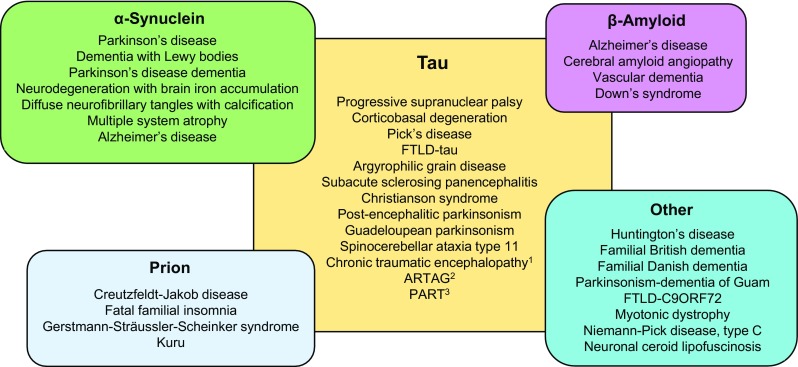
Tauopathies. Diagram illustrating the wide range of neuropathological conditions in which tau pathology is a significant feature. The *central panel* illustrates disorders in which tau pathology is the primary feature. The overlapping panels summarise conditions in which tau inclusions are accompanied by deposits of other disease-associated proteins [19, 358, 445, 469]. ^1^Chronic traumatic encephalopathy includes traumatic brain injury and dementia pugilistica; ^2^ARTAG, aging-related tau astrogliopathy includes globular glial tauopathy; ^3^PART, primary age-related tauopathy includes tangle-predominant dementia and clinically asymptomatic cases; FTLD, frontotemporal lobar degeneration

The heterogeneous group of dementias and movement disorders that comprise the neurodegenerative tauopathies are characterised neuropathologically by prominent intracellular accumulations of abnormal tau filaments that form neurofibrillary tangles, as well as other tau inclusions, in neurons and glia. Importantly, the discovery of multiple tau gene mutations in people with frontotemporal dementia exhibiting neuropathological evidence of FTLD-tau has shown that certain *MAPT* mutations result in abnormalities in tau protein that cause neurodegenerative disease [158]. These seminal findings paved the way for further investigation of the role of tau in cognitive dysfunction and neurodegeneration. However, tau neuropathology rarely exists in isolation, and hence, most tauopathies exhibit pathological abnormalities associated with the deposition of at least one other amyloidogenic protein, such as α-synuclein or huntingtin. This provokes the hypothesis that tau may have important pathological roles in these disorders with multiple pathologies (see Fig. 5) [194, 233]. This heterogeneity gives rise to a spectrum of tauopathy diseases with overlapping but distinct pathologies. The nature of the associated aggregated protein defines the neuropathological classification of the disease and may impact on the clinical symptoms that characterise each group of disorders, as summarised below.

### Tau in neurofibrillary tangles and amyloid pathology

Neuropathological Braak staging of AD brain characterises six progressive stages of disease propagation, which relate to the increasing severity of neurofibrillary tangle and amyloid plaque deposition in different brain regions [47]. The spread of AD pathology follows a consistent track through the brain, with neurofibrillary forms of tau appearing sequentially in the transentorhinal/peripheral cortex (Braak stage I), the CA1 region of the hippocampus (Braak stage II), limbic structures (Braak stage III), amygdala, thalamus and claustrum (Braak Stage IV), isocortical areas (Braak stage V), and finally, primary sensory, motor and visual regions (Braak stage VI) [46]. Based on the typical temporal-spatial progression of tau pathology in AD brain demonstrated by classical Braak staging, it has been proposed that affected brain regions are likely to be anatomically connected.

Structural biology studies have revealed that the dominant components of tangles in AD are paired helical and straight filaments, both types of filament being composed predominantly of abnormally phosphorylated tau protein [51, 261]. The actual molecular weight range of the six human CNS tau isoforms is 37–46 kDa (Fig. 1). However, on SDS-PAGE, tau in tangles extracted from AD brain resolves into three major bands with apparent molecular weights of 68, 64, and 60 kDa, with a minor band of approximately 72 kDa [176]. When dephosphorylated, tau from AD brain shows a similar band pattern to that of both dephosphorylated control adult human brain and recombinant human tau, with apparent molecular weights ranging from 48 to 67 kDa [190]. The reason for this discrepancy between the actual and apparent molecular weights of tau extracted from human brain is due to a combination of post-translational modification and variable SDS binding. Tangles from AD brain contain both 3R and 4R tau isoforms in a one-to-one ratio, similar to the isoform composition of tau in control adult human brain [163]. However, in other tauopathies, the form of tau deposited is characterised by the over-representation of either 4R or 3R tau isoforms. For example, PSP and CBD exhibit predominantly 4R tau, whereas insoluble tau in PiD is mainly 3R tau, and in FTLD-tau the isoform predominance depends on the specific disease-causing tau mutation [17, 105].

Together with tau deposition, the accumulation of Aβ as amyloid plaques in the extracellular space and around blood vessels is used to for the neuropathological diagnosis of AD at post-mortem [519]. In contrast to tau, Aβ deposition does not correlate with cognitive decline and plaque pathology exhibits a pattern of spread that differs from that of tau in AD brain [238]. A direct relationship between Aβ-mediated toxicity and tau pathology has repeatedly been proposed [38, 307], although understanding of the mechanisms that link Aβ and tau deposition is incomplete. However, it is clear from genome wide association studies, that some genetic risk loci for AD, such as apolipoprotein E (*APOE ε2, ε3,* or *ε4*) influence both amyloid and tau [108]. One hypothesis for the pathogenesis of AD proposes that the development of neurodegeneration in AD depends on Aβ working in concert with tau. Thus, elevated Aβ in transgenic mice overexpressing APP induces tau phosphorylation and intracerebral injection of Aβ into tau transgenic mice increases tangle pathology [148, 287]. Furthermore, immunising transgenic 3xTg-AD mice, which express mutant forms of tau, APP and presenilin 1, and develop both tangle and amyloid pathologies, with antibodies recognising Aβ reduces the amount of phosphorylated tau [376].

However, several studies have shown that cognitive decline is not the inevitable result of harbouring a considerable load of amyloid and tau pathology in the brain [257, 387, 438]. Aggregates gradual in AD correlates well with the number of tangles present; the demise of neurons far exceeds the degree of tangle pathology [147]. Furthermore, loss of synapses, potentially mediated by an as yet unidentified factor or mechanism, rather than the burden of AD pathology, best correlates with cognitive decline [325].

### Tau and α-synuclein pathology

Parkinson’s disease is a neurodegenerative disease affecting dopaminergic neurons. The principal pathological hallmark is the presence of Lewy bodies and Lewy neurites in the subcortical regions of the brain, which are composed of aggregated α-synuclein [464]. Hence, PD together with other α-synuclein related neurodegenerative disorders including Parkinson’s disease dementia, dementia with Lewy bodies, and multiple system atrophy, are collectively termed synucleinopathies [220, 463].

Notably, mutations in the *MAPT* gene cause variable extents of parkinsonism in affected people [215, 225]. These findings are supported by recent genome wide association studies, which have identified at least 24 genetic loci, of which the common genetic variants are associated with increased PD susceptibility [362]. Among these loci, the region encompassing the *MAPT* gene is one of the most significant hits, not only in rare familial cases but also in sporadic PD [270, 455]. It has been proposed that the H1 haplotype, one of two common genetic variations at the *MAPT* locus, may be related to the occurrence of “pure” tauopathy and may be linked to elevated amounts of tau in plasma [72, 393] and synucleinopathies [170, 492], whereas the alternate H2 haplotype correlates with reduced expression of tau protein and thus may have a protective effect [501]. Importantly, tau could also serve as a primary driver of parkinson-related neurodegeneration, independently of α-synuclein. Such a scenario exists in post-encephalitic parkinsonism, a 3R/4R tauopathy that may be attributed to post-viral encephalitis, in which α-synuclein pathology is absent [501], in PSP, and in parkinsonism due to specific *MAPT* mutations. Together, these findings raise the possibility that tau can function both as a risk factor and as a mediator of parkinsonism.

The co-occurrence of aggregated tau and α-synuclein in tauopathies and synucleinopathies has led to investigations of the interplay between tau and α-synuclein [222, 423]. Notably, Lewy bodies have been detected in more than half of the AD brains that come to autopsy and up to half of PD brains have sufficient tau and amyloid pathology for a neuropathological diagnosis of AD [222, 353]. The presence of neurofibrillary tangles containing tau in sporadic PD, has also been described [235, 439] and both tau and α-synuclein are enriched in synaptic fractions of brains affected by either tauopathy or synucleinopathy [357]. Additionally, pronounced tau pathology, including co-aggregation of tau and α-synuclein has been noted in familial Parkinson’s disease dementia [150, 401, 526]. Tau and α-synuclein colocalise in the same neuronal compartments, particularly in axons [124]. Moreover, tau fibrils are incorporated into Lewy bodies, colocalising with α-synuclein fibrils within individual aggregates [20, 223]. Further studies using mass spectrometry have also confirmed that tau is a component of Lewy bodies [232, 285] and increased phosphorylated tau has been shown to predict the rate of cognitive decline in PD [294].

In vitro studies have shown that co-incubation of tau and α-synuclein accelerates the fibrillisation of both proteins [159]. Tau expression also enhances the toxicity and secretion of α-synuclein and promotes the formation of smaller α-synuclein inclusions in human neuroglioma (H4) cells and primary neuronal cultures [26]. In turn, several studies have demonstrated that α-synuclein can promote tau phosphorylation. Nübling and colleagues showed that tau and α-synuclein can form detergent-resistant co-oligomers, and formation of these aggregates is enhanced by tau phosphorylation [324, 374]. An in vitro study showed that tau phosphorylation is facilitated by α-synuclein via PKA [233]. Moreover, α-synuclein enhanced GSK3β-mediated tau phosphorylation by formation of a tripartite GSK3β/α-synuclein/tau complex, resulting in the phosphorylation of tau at a number of residues [76, 247, 516]. Activation of MAPKs has also been reported in α-synuclein overexpressing transgenic mice, correlating with the presence of phosphorylated tau [138, 375]. Moreover, a recent study has shown that, the transcriptional regulator, tripartite motif-containing 28 (TRIM28), increases the amount of both tau and α-synuclein present in the nucleus, thereby increasing the toxicity of both proteins [423]. These findings suggest that in addition to the potential synergistic relationship between tau and α-synuclein they might also drive disease progression through shared mechanisms [423].

Together, these findings suggest that tau and α-synuclein interact to trigger formation of neuropathological lesions in the tauopathies and synucleinopathies. Events that increase the interaction of tau with α-synuclein could also modulate the activity of protein kinases and other tau modifying enzymes; thereby further influencing tau pathology and disease progression [353, 516].

### Tau in Huntington’s disease

Recent evidence has shown that tau is also involved in the neuropathology of Huntington’s disease, an autosomal-dominant movement disorder, in which cognitive decline is also a significant clinical feature [499]. HD is characterised biochemically by the presence of abnormal expansions of long polyglutamine tracts in huntingtin protein [269]. Increased amounts of total tau and phosphorylated tau, including rod-like deposits comprising mainly 4R tau, are evident in the brains of people with HD [128, 500]. A role has been proposed for huntingtin in the aberrant splicing of tau and the related microtubule-associated protein MAP2. The splicing factor SRSF6 accumulates in the striatum in HD and colocalises with nuclear inclusions bodies and other aggregates containing huntingtin [129]. Notably, SRSF6 is involved in the splicing of tau exon 10, which could provide an explanation for the deposition of 4R tau in inclusions in HD [128, 500]. Further evidence for the involvement of tau in HD comes from studies of animal models, including the R6/2 mouse, which overexpresses huntingtin exon 1 with an expanded polyglutamine repeat [64, 500]. R6/2 mice exhibit motor dysfunction and impaired learning and memory and intraneuronal inclusions of mutant huntingtin [99, 292]. Notably, increased tau phosphorylation is evident in the brains of R6/2 mice in parallel with reduced amounts of protein phosphatases [39, 174]. Increased tau phosphorylation has also been shown in HD, along with elevated GSK3 activity [268]. Taken together, these reports suggest a significant role for tau in the pathogenesis of HD.

## Tau-mediated neurodegeneration

Knowledge of the molecular mechanisms that underlie disease pathogenesis in the tauopathies is the subject of intense research. The following section focuses on the wide range of tau-related pathological events that occur at the molecular and cellular level during disease progression in the tauopathies. Alterations to the properties of tau that result from tau mis-splicing, aggregation, and post-translational modification, convert physiological forms of tau into pathological tau species that can cause tau to mislocalise in neurons. In addition, the detrimental effects of pathological tau may be amplified by dysfunction of multiple molecular pathways, including those involved in synaptic function, axonal transport, and protein quality control. Such pathological events could also act synergistically and elicit not only local cytotoxic effects but also fuel the intercellular spreading of tau pathology, and involve both neurons and glia.

### Tau gene dysfunction: mutations and splicing imbalance

P301L was the first mutation identified in the *MAPT* gene which resulted in tau dysfunction and neuronal death in FTLD-tau [213, 400]. Since then, a large number of mutations in *MAPT* have been reported to cause FTLD-tau, but notably, to date no mutations in *MAPT* have been associated with the development of AD [158]. Mutations in *MAPT* give rise to several different clinical phenotypes, the majority of which are frontotemporal dementia, but which also include Parkinson’s disease dementia, PSP, PD, AD, LBD, CBD, PiD, AGD, and FTD/amyotrophic lateral sclerosis (ALS). Certain *MAPT* mutations affect the ratio of 3R and 4R tau isoforms and increase tau phosphorylation [6]. Since 4R tau isoforms have a higher propensity to bind to microtubules, the presence of mutations can also have a significant influence on tau-microtubule binding [112, 311]. The influence of specific disease-associated mutations in tau on its functions beyond microtubule binding has yet to be well established. However, it is clear that *MAPT* mutations are detrimental to neurons and likely to impact on the conformation of tau, with resultant effects on its post-translational modification, interaction with other proteins, and a variety of intracellular processes.

### Tau aggregation

One of the most prevalent ideas about how tau contributes to the pathogenesis of tauopathies is that tau undergoes misfolding and oligomerisation into insoluble tau deposits. These tau aggregates gradually overburden neurons, affect fundamental cell functions and ultimately cause neuronal death [514]. Indeed, the appearance of tau deposits has been regarded as a typical pathological signature in many tauopathies, especially AD, and is used as an indicator of disease stage [47].

The structural basis of the aggregation propensity of tau lies in the two hexapeptide motifs located in the second and third microtubule binding repeats that display high β-sheet propensity and are further characterised as drivers of the abnormal self-assembly of tau [354, 355]. The hexapeptide motifs comprise tau residues 306–311 (PHF6, Val-Gln-Ile-Val-Lys-Tyr, VQIVKY) and 317–335 (PHF6*, Val-Gln-Ile-Ile-Lys-Tyr VQIINK) [380]. These regions of tau self-assemble in the absence of additional chemical stimuli [433, 498]. In vitro studies have demonstrated that PHF6 and PHF6* can form fibrillar aggregates in the presence of ammonium acetate [497]. PHF6 is located at the beginning of the third microtubule binding repeat and is present in all tau isoforms. In contrast, PHF6* is located at the beginning of the second microtubule binding repeat. Tau dimerisation can occur through interactions between two PHF6, two PHF6*, or between one PHF6 and one PHF6* motif [391]. Further recruitment of tau monomers and dimers could lead to the formation of a nucleation centre and once a critical cluster size is reached, tau oligomerisation can proceed in a dose and time-dependent manner [29]. Finally, tau oligomers elongate into protomers, which adopt a parallel, in register, cross β-sheet structure, typical of amyloid aggregates [332]. Ultimately, these tau filaments become the building blocks of neurofibrillary pathology in the tauopathies.

Although PHF6 and PHF6* motifs are prone to self-assembly, native tau is relatively resistant to aggregation. Hence, factors which enhance the assembly propensity of tau, or neutralise its charge, facilitate tau aggregation. Due to the presence of PHF6*, which is encoded by exon 10, 4R tau isoforms are more prone to aggregation than 3R tau isoforms. Mutations within the tau hexapeptide motif that enhance β-sheet propensity, such as the P301L tau mutation found in FTLD-tau, promote tau aggregation [288]. Conversely, introduction into these hexapeptide motifs of amino acid substitutions, such as proline residues, that disrupt β-sheet structure, render tau incompetent for assembly [55]. Notably, in addition to the increased aggregation propensity of exon 10, exons 2 and 3 also influence the kinetics of tau aggregation. The N-terminal insert encoded by exon 2 promotes tau aggregation, whereas expression of exon 3 exerts an inhibitory effect on tau aggregation in a process which is modulated by expression of exon 10 [540]. However, whether such effects result from changes in the overall charge of tau due to inclusion of the N-terminal inserts is unclear. Deletion of the positively charged Lys(K)280 residue, which is involved in localised electrostatic interactions, hinders tau self-assembly [497]. Phosphorylation of tau on serines, threonines and tyrosines, causes tau to become more negatively charged and tau acetylation neutralises positively charged lysine residues. Both of these post-translational modifications effectively reduce the overall positive charge on tau and can impact on tau folding. Furthermore, anionic condensing agents are well-documented as aggregation inducers. For example, heparin can bind to tau at multiple sites within the second and third microtubule binding repeats, as well as the flanking region and the N terminus, thereby stabilising assembly competent intermediates [267, 453]. Fatty acids, tRNA, and polyglutamic acid can also promote tau aggregation, although the regions of tau that bind these agents only partially overlap with those of heparin [517].

Neurofibrillary tangles have long been considered toxic to neurons. However, recent findings have challenged this view [92]. An in vivo model in which formaldehyde was used to treat primary hippocampal neurons showed that tau aggregates could induce apoptosis [363]. Toxicity was also observed in N2a mouse neuroblastoma cells in which expression of a fragment of mutant K18ΔK280 tau (Tau_258–360_, lacking K280) either alone, or together with full-length mutant tau (ΔK280) caused cytotoxicity [513]. The N2a cells expressing K18ΔK280 tau were positive for thioflavin S staining, implying that tau aggregation is closely associated with cytotoxicity. In contrast, findings from transgenic mice inducibly expressing P301L tau, demonstrated an improvement in memory, and neuronal loss was halted, when the mutant tau gene was switched off, despite tangle burden not being reduced [435]. Further studies showed that, tangle-bearing neurons appear to survive in inducible P301L tau-expressing mice, despite the apparent membrane disruption in affected neurons [104]. Whether tangles are toxic *per se* is still unknown, however, it is likely that tau species that are generated during the formation of tangles are damaging to cells. The precise nature of the tau species that result in neurotoxicity remain to be determined, but there is accumulating evidence that soluble oligomeric forms of tau, that may be generated during tangle formation, are damaging to neurons and to synaptic function [272, 465]. However, as discussed above, tau aggregation is affected by factors including mutation, isoform composition, and post-translational modification. Consequently, a variety of tau species with differing morphology, solubility, and disease-relevant properties can be generated. These differing forms of tau may form the molecular basis of distinct tau “strains” and might contribute to the wide degree of clinical and neuropathological heterogeneity observed in the tauopathies [432].

### Tau truncation

Proteolytic cleavage of disease-modifying proteins is found in a wide variety of human neurodegenerative diseases, including AD [151, 208, 373, 518], PiD [183, 343], CBD and PSP [16], transactive response DNA-binding protein 43 (TDP-43)-related FTLD [214], and PD [12], as well as polyglutamine diseases, such as Huntington’s disease [149].

The discovery of a protease-resistant core of tau within the paired helical filaments that comprise neurofibrillary tangles in AD brain was initially shown using a specific antibody that recognised a neoepitope generated by tau cleavage [165, 518]. These findings revealed that the core consists of tau fragments of 12 and 9.5 kDa, and the same antibody was shown to recognise tau protein that was C-terminally truncated at Glu391 (Table 2) [373]. The protease resistance of this 12 kDa form of tau led to the suggestion that truncation may be the mechanism that modifies tau such that it becomes prone to misfolding, adopting an abnormal conformation and self-assembling into filaments more readily than does full-length tau [372]. This view is supported in a study using DC11, a truncation-dependent conformational antibody, which recognises abnormal tau in AD brain but not tau in control brain [489]. Recombinant tau proteins truncated either at the N terminus or at both the N and C termini, are also recognised by DC11, indicating that both N- and C-terminally truncated tau species are present in tauopathy brain and can adopt pathological conformations [489]. Similarly, in vitro studies of tau aggregation have indicated that truncations occurring at Glu391 and Asp421, produce tau proteins that are more prone to aggregation than full-length tau [3, 33]. Together these findings provided the first in situ evidence that tau truncation might be a pathological mechanism in tauopathies.

**Table 2:** Tau fragments identified in human brain that may be involved in human tauopathies

Tau fragment	Amino acid residues	Mr (kDa)	Comments	References
C-terminally cleaved tau	M1-	40–53	Present in synaptosomes from AD brain C terminus not identified	[459]
Delta tau	H14/A15-D421		Associates with tangles in AD brain Identified in the brains of aged wild-type and transgenic 3xTg-AD and htau mice, which develop tangles, amyloid plaques and synaptic dysfunction. Induces tau filament formation and inversely correlates with cognitive function. Induced by Aβ in neurons and leads to apoptosis. Tau is cleaved at D13 by caspase-6 and at D421 by caspase-3	[30, 125, 151, 182, 208, 330, 369, 412]
NH2-tau	Q26-R230		Enriched in synaptosomal mitochondria in AD brain Induced by apoptosis in SHSY-5Y neuroblastoma cells. Present in hippocampus in AD11 transgenic mice which have chronic NGF deprivation during adulthood and display AD-like molecular and behavioural phenotypes	[7–10, 24, 89, 90, 404]
	E45-R230	17	Detected in AD, ALS, and control brain Generated in neurons by exposure to Aβ or by thapsigargin-mediated inhibition of autophagy. Induces neurodegeneration when expressed in mice. Not toxic when expressed in N2a or CHO cells, or neurons Generated by calpain-1 cleavage See related fragment A125-R230 below	[152, 271, 384, 414, 493]
	S129-(S71 in 0N3R tau)	33	Isolated from tangles in AD brain Decreased ability to bind to tubulin. C terminus not identified	[364]
	Q124-L441	43	Present in human brain Increased acetylation and detyrosination of tubulin when expressed in N1E-115 neuroblastoma cells	[109]
	A125-R230	17	Present in AD and control brain Not toxic when expressed in N2a or CHO cells, or neurons. Generated by calpain-2 cleavage See related fragment E45-R230 above	[152]
	I151-A391	29	Present in the neurofibrillary tangle core in AD brain Expression of either 3R tau_151–391_ (lacking 275–305) or 4R tau_151–391_ in transgenic rats induces tangle formation. Muscle weakness develops only in 4R tau_151–391_ rats	[132, 331, 373, 542]
Tau35	E187-L441	33–37	Present in AGD, PSP, and CBD, but not control brain Includes four microtubule binding repeats. Expression of Tau35 mice in transgenic mice induces tau pathology, cognitive and motor dysfunction	[16, 42, 216, 520]
Tau-CTF24	L243-L441	24	20–28 kDa C-terminal tau species detected in AD, CBD, PSP, and FTLD-tau, but not control brain Includes four microtubule binding repeats. Present in Tg601 mice which exhibit increased tau phosphorylation and synapse loss	[327]

Tau fragments that have been detected in human brain that is potentially associated with the development of tauopathy. The tau cleavage products are listed in order of their most N-terminal amino acid (single letter code). 3xTg-AD mice are transgenic for mutant forms of tau, amyloid precursor protein, and presenilin 1 [377]; AD11 mice are transgenic for NGF antibodies [63]; Tau35 mice are transgenic for the wild-type human tau fragment E187-L441 [42]; Tg601 mice are transgenic for wild-type 2N4R tau [240]

*3R* tau isoforms containing three microtubule binding repeats, *4R* tau isoforms containing four microtubule binding repeats, *Aβ* amyloid-β peptide, *AD* Alzheimer’s disease, *AGD* argyrophilic brain disease, *ALS* amyotrophic lateral sclerosis, *CBD* corticobasal degeneration, *CHO* Chinese hamster ovary, *FTLD* frontotemporal lobar degeneration, *PSP* progressive supranuclear palsy

Tau truncated at Asp421 colocalises with tangles in AD brain as well as in a number of transgenic mouse models of AD, indicating that the generation of this tau fragment may be an early event in tangle formation [30, 151, 208]. Similarly, expression of Tau_151–391_, including either three (Tau_151–391_3R) or four (Tau_151–391_4R) microtubule binding repeats, in the brains of transgenic rats induces neurofibrillary pathology that resembles human tauopathy [132, 262, 542]. Rats expressing either Tau_151–391_3R or Tau_151–391_4R exhibit pathological features including age-dependent increases in tau phosphorylation at multiple epitopes, and Gallyas-positive intracellular and extracellular tangles, which were positive for Congo red birefringence and thioflavin S [542]. Notably, extraction of sarkosyl-insoluble tau from Tau_151–391_ rat brain showed that these truncated forms of tau co-aggregate with endogenous rat tau [132]. These findings show that tau truncation facilitates misfolding of intact tau, which could be responsible for the generation of tangles in the brain in AD and related tauopathies.

Several other tau fragments have been described in a range of different tauopathies. An N-terminal neurotoxic tau fragment (Tau_26–230_) termed NH2-tau, has been detected in human SH-SY5Y cells undergoing apoptosis and also in the hippocampus of aged AD11 transgenic mice, which express antibodies to nerve growth factor and exhibit AD-like pathology, including Aβ accumulation and hippocampal-dependent memory deficits [89]. Tau_26–230_ is enriched in mitochondria isolated from AD synaptosomes [90], and this observation correlates with the altered function and quality control of mitochondria at synapses, as well as with synaptic dysfunction in AD [10]. Increased amounts of a 20 kDa C-terminally truncated tau fragment were present in synaptosomes from AD brain, compared to control brain [459]. A 33 kDa N-terminally truncated form of tau (starting at residue Ser71 in 0N3R tau, equivalent to Ser128 in 2N4R tau) was found in preparations of tangles purified from human AD brain [364]. A 17 kDa tau fragment (Tau_73–315_) was identified in cerebellar granule neurons undergoing apoptosis [62]. Interestingly, a different 17 kDa tau fragment (Tau_45–230_) was found in hippocampal neurons treated with Aβ [384] and also in post-mortem AD brain, and in a transgenic mouse expressing both human APP and tau [131, 414]. Overexpression of Tau_45–230_ induced apoptosis both in CHO cells and in neurons, and hence Tau_45–230_ has been proposed to have inherent neurotoxic properties [384]. However, these findings are controversial since others have reported this tau species to be smaller (11 kDa), to comprise residues Tau_125–230_, and to lack neurotoxicity [152]. Interestingly, Tau_45–230_ accumulates in lumbar and cervical spinal cord, as well as in upper motor neurons located in the precentral gyrus in ALS [493], suggesting that tau fragmentation may also have an important role in degeneration of motor neurons in ALS.

A 35 kDa C-terminal tau fragment (Tau_187–441_) lacking the N terminus of tau has been identified in neurodegenerative disorders characterised by overexpression of 4R tau isoforms, particularly in PSP [520]. Tau35 contains all four microtubule binding repeats and is highly phosphorylated in brains affected by tauopathy [520]. Minimal expression of Tau35 in transgenic mice is sufficient to cause several key features of human tauopathy, including aggregates formed of abnormally phosphorylated tau, progressive cognitive and motor deficits, and loss of synaptic components [42]. Similarly, another C-terminal tau fragment (Tau_243–441_), termed Tau-CTF24, was detected in Tg601 transgenic mice overexpressing wild-type human 2N4R tau [327]. Tg601 mice exhibit synapse loss in the nucleus accumbens and axonopathy in the ventral medial prefrontal cortex, as well as increased tau phosphorylation at the PHF1 epitope (phosphorylated Ser296/Ser404) in the striatum [240].

Tau cleavage could either generate fragments with a toxic gain of function, thereby switching on a cell death cascade, or alternatively such cleavage could induce and drive aggregation of tau and any associated disease-modifying proteins, leading to a loss of tau function. Supporting the latter scenario is the fact that truncated protein fragments can form the initial seeds required for aggregation and appear to be upstream in the proteopathic cascade that occurs in neurodegenerative disease [102, 132, 173, 214, 286, 542].

Along with the increasing number of tau fragments identified in cell and animal models of disease, increasing numbers of proteases that may be candidates for tau truncation have been identified. Proteases targeting tau include caspases, calpains, thrombin, cathepsins, asparagine endopeptidase (AEP), puromycin-sensitive aminopeptidase (PSA), human high temperature requirement serine protease A1 (HTRA1), and proteasomal proteases, which are described in more detail below [192, 508, 537].

#### Caspases

Caspases recognise at least four contiguous amino acids on their substrates, with an absolute requirement for an aspartate residue in the P1 position before the scissile bond [81]. Asp421 in tau is targeted by caspases-1, -3, -6, -7 and -8, generating tau fragments that are approximately 5 kDa smaller than full-length tau due to the removal of C terminus [151, 412]. In vitro, caspase-6 cleaves tau at Asp13 even more efficiently than the cleavage at Asp421, and these cleavage sites have both been validated by N-terminal protein sequencing or/and mass spectrometry [151, 208, 412]. Although activated caspase-6 has been found to colocalise with tau aggregates in AD brain, direct evidence of tau truncation at Asp13 in AD remains elusive [185]. Truncation of tau at Asp402, a putative caspase-6 cleavage site, has also been identified in transgenic animals, and Asp25 cleavage of tau, possibly due to the action of caspase-3 has also been detected in AD brain [185, 418]. However, to date, these sites have not been shown to be cleaved by any known caspases, at least in vitro [151]. Thus, only tau truncation at Asp421 by caspases has so far been validated both in vitro and in vivo and appears to be directly related to the development of tau pathology. Recently, Tau_26–230_, which has been reported to be neurotoxic in primary neuronal cultures, possible due to its effects on mitochondria, has also been found to be a product of caspase cleavage that is generated during apoptosis [10, 89, 90].

Further studies have identified a tau fragment cleaved at Asp421 by caspase-3 in COS and NTera-2 (NT2) cells transfected with human tau [125], in rodent primary cultured neurons [153], and htau [13] transgenic mice [369]. The presence of these caspase-cleaved tau products in AD brain was identified using antibodies TauC3 and α-ΔTau, which are specific for caspase-cleaved tau (Table 2) [151, 412]. TauC3 antibody also revealed consistent labelling of tangles and plaque-associated dystrophic neurites in the CA region of the hippocampus in human vascular dementia brain [100]. In addition, active caspase-3 colocalises with TauC3 labelling in plaques, blood vessels and pre-tangle neurons in AD brain [100]. Notably, cognitive decline and formation of tangles in aged wild-type mice also correlates with increases in caspase activity and caspase-3 truncated tau [330]. Similarly, in AD brain, caspase-6-cleaved tau fragments are associated with both pre-tangles and mature tangles, and these truncated forms of tau appear to correlate well with cognitive decline [151, 185, 208]. De Calignon and colleagues have shown that transient activation of executioner caspases in neurons of Tg4510 transgenic mice which inducibly express human P301L tau, leads to tau cleavage at Asp421 [102]. The resultant tau fragments generated by caspases exhibits tangle-related conformational epitopes, and thioflavin S-positive tangles [102]. Moreover, expression of Tau_151–421_ in hippocampal neurons leads to the induction of apoptosis, suggesting that caspase cleavage of tau at Asp421 might convert it into an apoptotic effector [125]. Tau_151–421_ also induces mitochondrial fragmentation and elevates oxidative stress in cells [66, 404]. Additionally, caspase-2 is also reported to cleave tau at Asp314 generating a N-terminal fragment. This fragment exhibited low propensity of fibrillation, but is able to infiltrate spines and dislocate glutamate receptors, causing synaptic dysfunction [539].

Notably, pseudophosphorylation of Ser422 can abolish in vitro tau truncation by caspase-3 at Asp421 [181] and can also enhance tau aggregation and impair axonal transport [476]. Together with the finding that phosphorylation of Ser422 in AD brain appears to precede truncation at Asp421 during neurofibrillary tangle maturation, this indicates that tau phosphorylation on Ser422 could inhibit tau cleavage by caspase in vivo [181].

#### Calpains

Calpains are cytosolic calcium-activated cysteine proteases, which exist as two major forms, calpain-1 and calpain-2 [166]. In addition to regulation by calcium, calpain activity is also negatively regulated by calpastatin, a calcium-dependent heat-stable calpain inhibitor. Protein cleavage by calpains is related only weakly to amino acid sequence and is more closely associated with polypeptide conformation [96, 479].

Increased calpain activity and depletion of calpastatin are observed in AD brain in comparison to age-matched controls [407, 427]. Several studies have shown that tau can be degraded by calpains in vitro [236]. Aβ treatment of cultured neurons leads to calpain activation and production of Tau_45–230_, suggesting that this tau fragment is generated by the action of calpain [384]. Highly phosphorylated insoluble tau in AD brain is less susceptible to calpain degradation than is soluble tau which has a lower phosphorylation state [293, 333], suggesting that phosphorylation may be linked to tau cleavage in vivo. However, calpain-mediated tau cleavage in AD brain may also be hampered by the conformation adopted by insoluble tau during its deposition in disease.

#### Thrombin

Thrombin is an extracellular serine protease generated by proteolytic cleavage of its precursor, prothrombin [127]. Thrombin has also been reported to be present in tangles in AD brain [5, 18], implying that it may be related to tau aggregation. Prothrombin mRNA is expressed in several regions of the rat and human nervous system [113], and both prothrombin and thrombin proteins are expressed in neurons [18]. It has been proposed therefore that thrombin could proteolyse tau in the brain, which is supported by the finding that in brain lysates incubated with different protease inhibitors, specific inhibition of thrombin in brain homogenates reduces tau degradation [15]. In vitro, tau is cleaved by thrombin at multiple arginine and lysine sites including Arg155, Arg209, Arg230, Lys257 and Lys340. The initial cleavage occurs at Arg155, producing a tau fragment of 37 kDa [513]. This truncated tau polypeptide is then subsequently cleaved at Arg230, yielding a 25 kDa tau fragment [378]. The resultant C-terminal tau fragment has a reduced capacity to promote microtubule assembly compared with full-length tau [378].

Phosphorylation of tau appears to make it more resistant to thrombin cleavage similar to the situation with caspases and calpains. Thus, thrombin cleavage of tau at Arg209, Arg230, Lys257 and Lys340 is suppressed by GSK3-mediated phosphorylation of tau at Thr212, Thr231 and Ser396/Ser404 and dephosphorylation of insoluble aggregated tau from AD brain causes it to become more susceptible to thrombin degradation [15]. PKA phosphorylation of tau also induces resistance to thrombin cleavage [506], supporting the view that phosphorylation may be a mechanism that dynamically modulates tau proteolysis.

#### Cathepsins

Several groups have shown in vitro that tau is cleaved by cathepsin D between amino acids 200 and 257, resulting in the generation of a 29 kDa tau species [31, 251]. Active cathepsin D and cathepsin B have been found in amyloid plaques in AD brain [65]. In human neuroblastoma cells inducibly expressing tau, disruption of lysosomes with chloroquine to releasing lysosomal proteases including cathepsins, results in inhibition of tau degradation and the appearance of tau aggregates [186]. In N2a cell expressing tau_RD_ΔK280, a tau fragment comprising the microtubule binding repeats but lacking Lys280, active cathepsin L generated amyloidogenic tau fragments, thereby indicating a role for cathepsin in tau aggregation [512]. In contrast with the degradation of tau by calpain, caspase-3 or thrombin, whereby tau phosphorylation suppresses proteolysis, tau degradation by cathepsin D appears to be accelerated by enhanced phosphorylation in vitro [251].

As cathepsins are primarily lysosomal proteases, an important question is how these enzymes could gain access to tau in neurons. One possibility is that inefficient translocation of tau or tau fragments across the lysosomal membrane could result in incomplete lysosomal cleavage of tau, generating small tau fragments [512]. In AD brain and under other conditions of cellular stress, cathepsin D and other proteases could contribute to tau proteolysis when the lysosomal system is disturbed [11, 42].

#### Asparagine endopeptidase

Another lysosomal cysteine proteinase, asparagine endopeptidase (AEP), has recently emerged as a tau protease. AEP degrades tau by cleaving it C-terminally at asparagine residues, abolishing the microtubule assembly function of tau and inducing its aggregation [537]. Notably, AEP is upregulated in human AD brain and in the brains of P301S tau transgenic mice. Knockdown of the AEP gene in P301S tau mice results in substantially reduced tau phosphorylation, rescue of synaptic function impairment and recovery of cognitive deficits. Furthermore, introduction of the N255A/N368A tau mutant, which abolished AEP cleavage at these two sites, also attenuated the pathological and behavioural defects in the P301S tau mice. Together with its recognition of APP as a substrate of AEP, these findings have resulted in the suggestion that AEP could be a useful target for therapeutic intervention in the tauopathies [538].

#### Puromycin-sensitive aminopeptidase

Puromycin-sensitive aminopeptidase (PSA) is found in neurons, but not in surrounding glial cells or in blood vessels [478] and comprises over 90% of the aminopeptidase activity in the brain [328]. PSA can digest tau isolated from brain tissue in vitro and expression of PSA is inversely correlated with vulnerability to tau pathology [244, 443]. In *Drosophila* expressing human tau, PSA expression reduced the amount of tau and protected against tau-induced neurodegeneration, whereas flies expressing a PSA loss-of-function mutant exhibited exacerbated neurodegeneration [244]. Hence, PSA could modulate the amount of tau present in the brain. Interestingly, in FTLD-tau brain tissue, expression of PSA is elevated fivefold in the cerebellum compared with the frontal cortex [244]. This finding, combined with the observation that the cerebellum is less affected than cerebral cortex in the tauopathies [77], reinforce the potential protective role of PSA against neurodegeneration.

#### Human high temperature requirement serine protease A1

Human high temperature requirement serine protease A1 (HTRA1) is a secreted ubiquitously expressed, ATP-independent serine protease with intrinsic disaggregating activity [78]. Mutations in HTRA1 are associated with the development of age-related macular degeneration and small vessel disease, and recently HTRA1 has been shown to colocalise with tangles and plaques in AD brain [175, 193]. There is an inverse correlation between HTRA1 and plaque and tangle numbers in AD brain and in keeping with this total amount of tau and phosphorylated tau inversely correlate with HTRA1 in AD, but not in control brain [475]. HTRA1 can degrade both soluble and aggregated tau at multiple sites, producing a range of small tau fragments ranging from 9 to 22 residues in length [475]. Little is known regarding the consensus sequences required for HTRA1 cleavage, although cleavage after the hydrophobic amino acids Val, Leu, and Ile are preferred sites in tau. HTRA1 appears to preferentially target N- and C-terminal regions of aggregated tau, cleaving tau within the microtubule binding domain [395]. Due to its intrinsic ability to solubilise misfolded proteins, HTRA1 can both disaggregate and proteolyse tau. This ability has recently been demonstrated in HEK293T cells expressing P301L tau aggregates, in which HTRA1 was able to solubilise tau and to enhance its degradation [395]. Furthermore, when tau was exogenously expressed in PC12 cells colocalisation of HTRA1 and tau with microtubules was demonstrated, alongside increased HTRA1 mRNA and HTRA1 activity [475]. Thus, HTRA1 could target aggregated tau and potentially limit the spread of tau pathology in the tauopathies by inducing its cleavage and clearance [395].

#### The ubiquitin-proteasome system

The ubiquitin-proteasome system (UPS) regulates protein quality control in both the cytoplasm and the nucleus by eliminating damaged, misfolded, and mutant proteins [279]. Blocking the activity of the proteasome catalytic core inhibits tau degradation in SH-SY5Y cells expressing exogenous human tau [98]. Furthermore, in vitro studies have shown that the proteasome degrades unfolded recombinant tau in an ubiquitin-independent manner, generating stable tau intermediates of approximately 27 and 17 kDa [98, 510]. In AD brain, proteasome activity is decreased, which could contribute to the accumulation of protein aggregates, including tau filaments [250, 428].

### Axonal transport impairment in the tauopathies

Besides regulating microtubule dynamics, tau regulates the axonal transport of proteins and organelles by influencing the motor proteins dynein and kinesin. Whilst dyneins transport cargoes towards the minus ends of microtubules, directing them to the cell body, the majority of kinesins transport cargoes towards the plus ends of microtubules in the direction of the axon terminus [106, 326]. Tau can dynamically regulate the function of the axonal transport machinery through multiple mechanisms [115, 120, 485].

Axonal and cell body accumulations of organelles and other proteins frequently occur in neurodegenerative disease, leading to the appearance of axonal swellings and spheroids [337]. Such pathologies suggest that defective functioning of axonal transport may contribute to disease. Axonal transport requires intact microtubules, functional motor proteins, correct cargo attachment to motors, and sufficient ATP, supplied by mitochondria. Thus, each of these four components of the axonal transport system can be a target of pathogenic proteins [106]. Indeed, axonal transport defects have been described as an early pathological feature in a variety of animal models of AD and tauopathies [28]. Neurons containing tangles exhibit severely impaired anterograde transport along axons as well as in basal dendrites and impaired retrograde transport in apical dendrites [106, 337].

Recent work supports the idea that tau affects axonal transport by both compromising the structure of microtubules, and disrupting components of the axonal transport machinery. Abnormal modification of tau, such as increased phosphorylation, truncation and acetylation, impairs the interaction of tau with microtubules and the ability of tau to stabilise microtubules [351, 480]. Moreover, pathological forms of tau have a reduced ability to promote microtubule assembly and form an organised cytoskeletal network [161]. Furthermore, overexpression of mutant or wild-type tau in mice results in dendritic missorting of tau and destabilisation of microtubules, an effect that can be rescued by microtubule-stabilising drugs [536]. Mislocalisation of tau to dendrites is a neuropathological feature of AD brain which occurs early during disease pathogenesis, possibly even pre-clinically, and prior to tau aggregation [45, 74]. Loss of tau function therefore leads to a loss of the microtubule tracks required for efficient axonal transport. In addition, reduced tubulin acetylation has been observed in neurons containing tangles in AD brain [198], indicating that tubulin acetylation could also be involved in impairing axonal transport.

Tau also interferes with binding of the molecular motor proteins dynein and kinesin, to microtubules. Tau reduces the binding frequency as well as the mobility of these two proteins, slowing both anterograde and retrograde transport [441]. Overexpression and mislocalisation of tau modulates kinesin-based transport by directly inhibiting the access of these motors to microtubule tracks [120]. Moreover, in vitro studies have revealed that tau inhibits kinesin-mediated transport, not only by reducing the distance travelled by individual kinesins but also by reducing their velocity [115, 120, 466]. Tau reduces the number of motors that are engaged with cargoes and thereby interferes with axonal transport of cargoes [491]. Protein levels of both the kinesin motor-mediated axonal transport machinery and of the dynein-mediated retrograde transport machinery are reduced in AD [346]. Such reductions, especially of kinesin light chain and dynein intermediate chain compromise the capacity of these motor proteins. Tau sequesters the available kinesin, and thereby limits axonal transport of other cargoes [258, 485] and regulates the release of cargo vesicles from kinesin chains by activating PP1 and GSK3β [242]. Thus, increased activation of GSK3β contributes to transport deficits by aberrant phosphorylation of light chain of kinesin, resulting in premature release of kinesin from its cargoes [348]. It was further found that tau mislocalises the kinesin adapter-molecule C-Jun amino-terminal kinase (JNK)-interacting protein 1 away from microtubules and into the neuronal soma [225, 227]. Notably, a recent report has suggested that, at least in *Drosophila*, loss of tau results in inhibition of kinesin-driven axonal transport leading to the accumulation of synaptic proteins in the neuronal cell body and subsequent synaptic decay [496]. The molecular mechanism underlying the functional deficit appears to be mediated by JNK activation caused by microtubule instability upon loss of tau function [496]. Consequently, pathological tau cannot only compromise the structural basis of synapses, but also inhibit transport of other cargoes to the synapse, resulting in synaptic degeneration. In such a scenario, displaced organelles, such as mitochondria may accumulate in the neuronal soma, resulting in energy deprivation and oxidative stress which fuels the progression of pathology and neuronal demise in AD and related disorders.

### Nuclear tau dysfunction

Key events involved in nuclear tau dysfunction include tau mutation, tau abnormal phosphorylation and oxidative stress. In fibroblasts and lymphocytes from FTLD-tau affected patients, a series of cell deficits are observed in cells bearing tau mutants, including increased susceptibility of the cells to stress, altered gene transcription, and chromosome aberrations [421, 422]. In contrast, the impact of phosphorylation on the nuclear function of tau is more complex. On one hand, there is evidence showing that abnormal phosphorylation of tau, such as is apparent in human tauopathies, reduces the nuclear translocation of tau [282] and the ability of tau to bind and protect DNA [61, 312, 403]. These results suggest a detrimental loss-of-function of nuclear tau upon its increased phosphorylation. The absence of nuclear tau enhances oxidative stress-induced DNA and/or chromosomal damage. However, a gain of toxic function for highly phosphorylated tau in the nucleus cannot be excluded. It has been suggested that increased tau phosphorylation lies upstream of oxidative stress-induced DNA strand breakage [139, 344, 494]. Moreover, accumulation of phosphorylated tau in the nucleus triggered by Aβ exposure and by viral infection has also been suggested [344]. Phosphorylated tau in the nucleus may be recruited to stress granules by TIA1, altering granule dynamics and sensitising cells to stress [53]. Downstream, the outcome of nuclear tau dysfunction in disease could include (1) disrupted heterochromatin organisation, leading to cell cycle re-entry which is fatal to neurons [446], and (2) dysregulated gene expression and rRNA synthesis, giving rise to altered protein synthesis [139, 199]. Notably, tau aggregates have also been found in the nucleus in affected neurons in Huntington’s disease, FTLD-tau, and AD [128, 130, 335]. However, the consequences of harbouring aggregated tau in the nucleus in relation to tauopathy, await further investigation.

### Dendritic tau in the tauopathies

A few reports have highlighted a gain of toxicity of dendritic tau in promoting neurodegeneration under pathological conditions [80]. Ittner and colleagues showed that dendritic tau mediates Aβ toxicity by targeting the non-receptor-associated tyrosine kinase Fyn, to post-synaptic *N*-methyl-d-aspartate receptors (NMDARs) in mouse brain [226]. Fyn kinase then phosphorylates the NR2B subunit of the NMDAR, rendering neurons susceptible to excitotoxicity mediated by Aβ [361, 413, 420]. Furthermore, tau directly binds to Fyn [273, 276, 483]. Both trafficking of Fyn into post-synaptic sites in dendrites and stress-induced dendritic atrophy are abolished in tau knockout mice [226, 310]. Dendritic tau has also been shown to form a complex with post-synaptic density (PSD)-95, suggesting that tau can act as a synaptic scaffolding protein [67, 226, 345]. However, this notion is controversial because interactions between tau and PSD-95 can be protective [360]. Exposure to Aβ results in tau mislocalisation to the somatodendritic compartment, mediates AMPA receptor signalling deficits in APP_swe_-transgenic mice, which express the familial AD-associated APP mutation KM670/671NL [338]. APP_swe_ mice exhibit enhanced Aβ production and the formation of amyloid plaques along with cognitive deficits [209]. There is also evidence that in rTg4510 mice, mislocalised dendritic tau is sufficient to perturb AMPA and NMDA receptor signalling, leading to synaptic dysfunction [205].

Another route through which dendritic tau can exert toxicity relates to the interplay between tau and the microtubule severing enzymes, katanin and spastin, both of which induce microtubule depolymerisation [326]. The presence of tau in axons protects microtubules from severing by katanin. In contrast, dendritic tau recruits tubulin tyrosine ligase-like 6 (TTLL6), causing it to mislocalise to dendrites, where it polyglutamylates the microtubules, increasing their susceptibility to spastin cleavage [486, 532]. Mislocalisation of tau in dendrites results in the loss of normal microtubule structure and deleterious effects on axonal transport [534]. Therefore, increased tau in dendrites causes cargoes, such as mitochondria, vesicles, and neurofilaments that are normally transported between the somatodendritic and axonal compartments and nerve terminals, to become mislocalised in affected neurons [532, 534].

Although some components of the mechanism underlying the toxicity of dendritic tau have been identified, the upstream events leading to tau missorting are less well understood. Several studies indicate that Aβ-induced tau mislocalisation is permissive for the deleterious effects of Aβ [67, 533, 535]. More recently, it has been shown that the physiological translocation of tau from dendrites to the post-synaptic density is reduced following Aβ exposure, resulting in tau accumulation in dendritic spines [136]. However, the finding of dendritic tau in AD brain regions that do not have significantly elevated Aβ [48] raises the question of whether tau mislocalisation is necessary and sufficient for Aβ toxicity [38, 80].

The accumulation of tau harbouring the FTLD-tau mutation P301L, in dendritic spines has led to speculation that tau mutations contribute, either directly or indirectly, to tau mislocalisation [205, 524]. Recently, a study using fluorescence recovery after photobleaching revealed that the axodendritic gradient distribution of tau is inverted by overexpression of either wild-type or mutant P301L tau, suggesting that the protein level of tau may also be a modulator of tau dendritic mislocalisation [523]. Several lines of evidence suggest an association between tau post-translational modifications and its somatodendritic redistribution [80]. Phosphorylation of tau within the KXGS motifs located within the microtubule binding domain dramatically reduces the ability of tau to bind to microtubules [179, 336], which could be one of the initial steps involved in tau mislocalisation, as described above. Correspondingly, activation of MARK or AMPK, both of which phosphorylate tau at KXGS motifs, is critical for the synaptotoxicity and dendritic spine abnormalities induced by Aβ [180, 318, 530]. Phosphorylation in the proline-rich domain of tau, particularly at Ser202/Ser205, may also contribute to its dendritic localisation. In AD, mislocalised dendritic tau is phosphorylated at Ser202/Ser205 but not at either Ser396/Ser404 or Thr231/Ser235 [234, 535]. Phosphorylation of Ser202/Ser205 is associated with activation of MARK and Cdk5 but not GSK3β. Conversely, pseudo-phosphorylated tau at Thr231/Ser235, Ser262/Ser356 and Ser396/Ser404 markedly enhances the targeting of tau to spines [524]. Furthermore, newly synthesised tau is missorted to the somatodendritic compartment prior to its phosphorylation by MAPK [532]. Taken together, the link between tau phosphorylation and mislocalisation is evident, whereas the spatial and temporal relationship between these two events is yet to be established. Notably, tau acetylation should also be considered as being a putative factor in tau mislocalisation in neurons. Acetylated tau also has an impaired ability to bind to microtubules [83] and pseudo-acetylated tau has recently been found to missort into the somatodendritic compartment, which could be related to the observed perturbation of the axon initial segment cytoskeleton in the animal models of AD [195, 458].

### Tau and mitochondrial dysfunction

Mitochondrial dysfunction has been suggested to play a critical role in the development of tauopathy [528]. Accumulation of tau disrupts mitochondrial localisation in human tauopathy brain and in animal models of disease, such as those expressing tau mutations associated with FTD [97, 259]. For example, increased reactive oxygen species have been reported in transgenic P301L tau mice [97, 259]. Although overt effects on mitochondrial dynamics have not been observed in neurons cultured from P301L tau knockin mice, expression of this tau mutation significantly reduces the number of mitochondria in axons [417]. The findings in P301L tau knockin neurons of increased volumes of individual motile mitochondria, accompanied by decreased phosphorylation of endogenous tau, suggest a role for tau and tau phosphorylation in the regulation of mitochondrial function and/or biogenesis [417].

Further evidence in support of a role for tau in maintenance of mitochondrial function comes from studies of the relationship between tau and the mitochondrial fission protein, dynamin-related protein 1 (Drp1). Interaction between Drp1 and phosphorylated tau increases fragmentation of mitochondria, resulting in mitochondrial deficiency in affected neurons [320]. Increased Drp1 and mitochondrial fragmentation have also been reported in mice overexpressing tau and GSK3β [415]. Conversely, reducing Drp1 expression decreases phosphorylated tau and reduces mitochondrial dysfunction in P301L tau over expressing mice [243]. In AD, there is evidence for both increased fission and decreased fusion of mitochondria, as well as enhanced interaction of Aβ with Drp1, impaired axonal transport of mitochondria, and synaptic degeneration [319].

Notably, several groups have demonstrated an association between mitochondria and an N-terminal tau fragment (Tau_26–230_), detected in cellular and animal models of AD, as well as in AD brain [8, 24, 404]. Tau_26–230_ is enriched in mitochondria prepared from AD brain, correlating with synaptic and mitochondrial dysfunction [8]. Tau_26–230_ is also associated with Parkin leading to increased Parkin-dependent turnover of mitochondria, and neuronal death which could be partially restored by suppressing mitophagy [90]. A recent report has also indicated that tau truncated at Asp421 induces mitochondrial fragmentation possibly through a reduction in optic atrophy protein 1 [389]. Increases in cytochrome c oxidase IV, translocase of outer mitochondrial membrane 20, and mitochondrial DNA, which are indicators of mitophagy, have also been detected in AD brain and in tau transgenic mice [210]. Furthermore, overexpression of tau has recently been shown to result in defective mitophagy in neurons, along with accumulation of tau in the outer mitochondrial membrane and consequent increases in mitochondrial membrane potential [210]. Taken together, these findings suggest that tau and Aβ significantly affect mitochondrial integrity and the maintenance and function of synapses in health and disease.

### Tau clearance by the ubiquitin-proteasome system and autophagic-lysosomal degradation

Incomplete clearance from the brain of pathological tau could also result from its inefficient degradation through the UPS and/or autophagic-lysosomal system. The UPS mediates the selective degradation of nuclear and cytosolic proteins, whereas the autophagy-lysosomal system is primarily involved in the clearance of long-lived proteins and organelles through non-selective bulk degradation [424].

Several studies have demonstrated that tau can be degraded through the UPS and by the autophagic-lysosomal system. Identification of ubiquitination sites on both soluble and insoluble highly phosphorylated tau has provided a strong evidence for the role of the UPS in tau clearance [279]. Moreover, unfolded tau is proposed to be processed independent of ubiquitination [98].The significance of the UPS in tau clearance is further supported by the identification of UPS components, such as heat shock protein 27 and CHIP as tau binding partners [279]. Thus, it is not surprising that dysfunction of the UPS is observed in a number of tauopathy models and in AD [107, 359]. Phosphorylated tau aggregates bind to the 20S subunit of the proteasome and this could interfere with tau degradation by inhibiting proteasomal activity [249]. Although it has not yet been established whether damage to the UPS precedes or is induced by tau aggregate formation, manipulation of the UPS may be a potential treatment strategy in the tauopathies. For example, activating the 26S proteasome via the cAMP-PKA pathway enhances tau degradation and rescues the damaging effects of tau oligomers on UPS activity [308, 359]. In contrast, the results of targeting the HSP response are more variable possibly due to the differential selectivity of HSPs. Thus, induction of HSP70 reduces tau aggregation [117, 392], whereas inhibiting HSP90 yields similar beneficial effects [111, 314]. In both cases, the role of CHIP is pivotal for tau degradation [392, 452].

Whereas soluble tau is preferentially degraded by the proteasome, pathological forms of phosphorylated tau appear to be directed towards to the autophagic-lysosomal system for disposal. Indeed, direct evidence for autophagy as the primary route for clearing phosphorylated, but not endogenous, tau has been obtained from monitoring the differential degradation rates of phosphomimic tau mutants, wild-type tau and endogenous tau in neurons [264, 416]. It is not unreasonable to propose that malfunction of the autophagic-lysosomal system could contribute to the development of tauopathy. Indeed, impaired autophagy has been repeatedly reported in tau-mediated neurodegenerative diseases. For example, accumulation of immature autophagic structures and intermediates, such as autophagosomes and late autophagic vacuoles, has been observed in dystrophic neurites in AD brain, and in animal and cell models of AD, suggesting impaired degradation of autophagic vacuoles by lysosomes [303, 367, 471]. Additional evidence of a role for autophagy in AD comes from the colocalisation in neuronal and glial cells of Alz-50 antibody immunoreactivity, an early indicator of tau misfolding with lysosomes [217, 218]. Furthermore, both inhibition of autophagosome formation and perturbation in lysosomal function, were found to account for delayed degradation of tau, enabling its accumulation in human neuroblastoma cells and transgenic mice [42, 186]. Stimulating mTOR activity, which represses autophagy, also increases total and phosphorylated tau in P301S tau mice [56]. Autophagy deficiency also results in the formation of intracellular inclusions of phosphorylated tau in autophagy-related protein 7 (Atg7) knockout mice [219]. Moreover, genetic ablation of cathepsin D enhances neurotoxicity and reduces lifespan of *Drosophila* [31, 252]. In contrast, stimulation of autophagy promotes tau clearance, reduces tau aggregation and cytotoxicity, and rescues neurodegeneration [32, 85].

Tau fragmentation also impacts on tau degradation. Expression of N-terminally truncated tau in Tau35 mice is associated with dysfunction of autophagy/lysosomal degradation [42], and caspase-3-mediated truncation of tau at Asp421 enhances autophagic rather than proteasomal degradation of tau [116]. When expressed in N2a cells, tau_RD_ΔK280, the repeat domain of tau with a K280 deletion, which itself has a propensity to aggregate, was degraded by autophagy generating highly aggregation-prone products [512].

The mechanisms underlying the preferential degradation of pathological forms of tau by autophagy are unclear, although highly phosphorylated and truncated tau is both susceptible to aggregation. Accumulation of tau oligomers could exceed the capacity of the UPS to clear them from neurons or the UPS could be directly inhibited by disease-associated tau aggregates [249]. Tau cleavage may also remove its polyubiquitination site which would prevent or limit clearance by the UPS and could expose motifs in tau that are targeted by chaperone-mediated autophagy [82, 512]. Impaired autophagy, due to defective microtubule-associated autophagic vacuoles, could also result in p62/SQSTM1 accumulation which might sequester other proteins required for proteasomal degradation [40, 303]. Collectively, these findings serve to highlight the pivotal role of autophagy in disease pathogenesis in the tauopathies, and suggest that restoration of efficient lysosomal proteolysis and autophagy offer a promising therapeutic strategy.

### The unfolded protein response and tauopathy

The unfolded protein response (UPR) is elicited by the endoplasmic reticulum (ER) to internal and external insults, including protein misfolding. Initiation of the UPR results in signalling through three branches, each of which utilises one of the three ER stress sensors: inositol-requiring transmembrane kinase/endonuclease 1 (IRE1), activating transcription factor 6 (ATF6), or (PKR)-like endoplasmic reticulum kinase (PERK) [437]. Initiation of the UPR then triggers signalling cascades, which lead to different outcomes, depending on the signalling branch activated. For example, activation of IRE1 initiates the splicing of X-box binding protein 1 (XBP1) mRNA, leading to a frame-shift and expression of spliced X-box-binding protein 1 (sXBP1), which drives transcription of genes including ER chaperones which facilitate protein folding in the ER [419, 447]. PERK is a transmembrane protein kinase that phosphorylates and activates eukaryotic initiation factor 2α (eIF2α). Activated eIF2α blocks the loading of mRNA to ribosomes during the initiation of transcription, leading to reduced protein synthesis [126] and preferential translation of activating transcription factor (ATF) 4. In parallel, UPR activation causes the cytoplasmic domain of ATF6 to be released from the ER, cleaved and translocated to the nucleus. Ultimately, these ATFs modulate the expression of an array of genes governing ER protein folding capacity, autophagy, redox control, amino acid metabolism, and apoptosis, including CCAAT-enhancer-binding protein homologous protein (CHOP) [200].

Accumulating evidence from genetic and biochemical studies has shown that the UPR is activated at early stages in tauopathy brain [206, 366, 482]. UPR activation has also been implicated in cell and animal models of tauopathy, as well as in torpor, a physiological in vivo model of hypometabolism [487], although the means by which tau contributes to the activation of the UPR remains unknown. Accumulation of P301L tau in transfected HEK cells facilitates the interaction of tau with ER membrane and with proteins essential for ER-associated degradation (ERAD), resulting in UPR activation [2]. In JNPL3 mice, accumulation of transgenically expressed P301L tau in the rough ER increases its contacts with mitochondria which may potentially disrupt calcium homeostasis [390]. Indirect mechanisms, such as generation of reactive oxygen species and impaired protein degradation caused by microtubule disorganisation may also contribute to UPR activation [303]. In vitro induction of ER stress also correlates with Aβ oligomer-induced tau phosphorylation [409].

Several studies have highlighted the role of GSK3β-mediated tau phosphorylation in UPR activation and tauopathy progression. In AD brain, increases in UPR markers closely correlate with the presence of phosphorylated tau and GSK3β [366]. In AD hippocampal neurons harbouring abnormally phosphorylated tau, phospho-PERK colocalises with both GSK3β and phosphorylated tau [207]. Activation of PERK facilitates P301L tau phosphorylation, which is reduced by a PERK inhibitor in rTg4510 mice [405]. Induction of the UPR in HepG2 and SHY-SY5Y cells also correlates with increased activity of GSK3β [253, 460]. Correspondingly, inhibiting GSK3β with lithium chloride protects tau from the increase in phosphorylation induced by thapsigargin, both in vitro and in rat brain [142]. Furthermore, it has been suggested that there may be a vicious cycle wherein UPR activation contributes to tau phosphorylation and that increased tau phosphorylation also activates the UPR. Elevations in active PERK and eIF2α, splicing of XBP1 mRNA, and elevated CHOP mRNA have been found in primary neurons treated with the protein phosphatase inhibitor, okadaic acid, which also increases tau phosphorylation [202]. Increasing the expression of selenoprotein S, a component of an ER membrane complex that removes misfolded proteins from the ER decreases tau phosphorylation induced by ER stress [425]. Initiation of the UPR can enhance GSK3β-mediated tau phosphorylation through different mechanisms. First, activated UPR sensors, particularly IRE1 and PERK, can either inhibit Akt or suppress insulin-induced inhibition of GSK3β, leading to increased GSK3β activity [133, 305]. Second, the ER-associated chaperone, binding immunoglobulin protein (BiP), which is elevated by UPR activation, facilitates tau phosphorylation through enhancing the association of GSK3β with tau [305]. Overexpression of SIL1, a co-chaperone of binding immunoglobulin protein (BiP), significantly reduces tau phosphorylation induced by elevated expression of BiP or thapsigargin treatment [304]. Finally, UPR activation increases the activity of GSK3 in vitro by selective removal of inactive GSK3 [365]. Inactive GSK3 also accumulates in lysosomes in tauopathy brain. Additionally, activation of the UPR and induction of eIF2α activity could contribute to neurodegeneration by repressing global protein translation, potentially attenuating the synthesis of several key synaptic proteins leading to synapse dysfunction and cognitive decline. In support of this speculation, the hippocampal amounts of the GluR2, AMPA receptor subunit, PSD-95, and synapsin-1, all decreased following tunicamycin-induced UPR activation in rats [291]. Collectively, these findings indicate that modulation of the UPR, particularly the PERK/eIF2α signalling branch, may exert a dual beneficial effect in the tauopathies, not only by restoring vital protein synthesis in compromised neurons, but also by decreasing tau phosphorylation.

### Tau seeding and propagation

A key characteristic of prion-like protein transmission is that of intercellular propagation, which results in the spread of disease-related protein aggregates across the brain [137, 402]. Tau has been described as having prion-like properties because in P301L tau transgenic mice, in which tau expression was restricted primarily to the entorhinal cortex, misfolded tau spreads from the entorhinal cortex into the CA1 region of the hippocampus and granule cells of the dentate gyrus [103, 302]. Tau aggregation and synaptic degeneration were observed in neurons lacking detectable expression of P301L tau. Thus, the mutant transgenic tau-induced deleterious aggregation of both endogenous wild-type and transgenically expressed tau [103]. Subsequent studies have shown that injection of brain extracts from P301S tau transgenic mice or human tauopathy into the brains of mice overexpressing wild-type human tau, results in tau aggregation not only around the injection site, but also in more distal, connected brain regions [4, 79]. These findings suggest that neuronal connectivity, rather than proximity, is important for the spread of tau pathology. As well as demonstrating the ability of tau to undergo “prion-like” propagation from an initial restricted source, these studies also imply that pathological forms of tau are transmitted *trans*-synaptically. Further evidence in support of tau *trans*-synaptic propagation has been obtained from cell models. For example, tau aggregates released from HEK293 donor cells are taken up by hippocampus neurons, and this process is significantly enhanced by the formation of presynaptic contacts between neurons [58]. There is also evidence showing that tau can be secreted, transmitted, and taken up through cellular structures other than synaptic connections, suggesting the existence of “*trans*-cellular” propagation pathways [103, 385].

Tau secretion may be mediated through several different mechanisms, including unconventional secretion, ectosomal and exosomal release, and/or tunnelling nanotubes. Under physiological conditions, tau in cultured rat cortical neurons is released into the medium and stimulating neuronal activity enhances release of tau, the bulk of which is non-vesicular through a calcium-dependent mechanism [398, 522]. Tau release from neurons occurs in the absence of cell death, indicating that under these conditions the presence of extracellular tau is not the result of neuronal dysfunction [398, 522]. Enhanced neuronal activity also increases the steady-state level of extracellular tau in brain interstitial fluid in wild-type mice [525]. Similarly, release of tau from both HEK293 cells inducibly expressing 2N4R tau, and differentiated induced pluripotent stem cell-derived human neurons expressing 3R tau, is mediated through an unconventional, temperature-dependent mechanism that is not associated with vesicle secretion [68]. In human neuroblastoma M1C cells inducibly expressing 0N4R tau, and in rTg4510 mice, secretion of tau is also mediated in part by exosomes which display a propensity to seed aggregation of endogenous tau [396, 429]. It appears that, although a small proportion of tau is released in exosomes and ectosomes [119, 429], the majority of tau released from neurons under physiological conditions is not surrounded by a lipid envelope [397]. However, a recent study in P301S tau mice has shown that microglia-derived exosomes may be responsible for transduction of tau between neurons [22]. Recently, tunnelling nanotubes have been identified as another mechanism by which tau aggregates may be transmitted through direct contact between neurons. Increased numbers of tunnelling nanotubes are detected on neurons following exposure to exogenous tau [472]. Tau release from neurons due to chaperone-dependent exocytosis has also been identified [135]. The presynaptic co-chaperone cysteine string protein-alpha (CSPα) is involved in the release of several aggregated proteins associated with neurodegenerative disease through a non-canonical exocytosis pathway [135], and CSPα is also dysregulated in AD [477]. Interestingly, both knockout of CSPα and increased proteasomal degradation of CSPα, result in neurodegeneration in vivo suggesting that CSPα may have a protective role [430, 448, 449].

The concept of extracellular tau suggests additional roles for aggregated tau, which may be dependent on its uptake by adjacent and/or connected neurons. Low molecular weight aggregates and short fibrils of recombinant tau can be internalised by endocytosis [521]. Furthermore, extracellular AD brain-derived tau aggregates have been reported to be endocytosed by both HEK293T non-neuronal cells and SHSY5Y human neuroblastoma cells [140, 434]. In cultured cell lines, primary neurons and wild-type mice, extracellular tau attaches to heparan sulfate proteoglycans (HSPGs) and thereby enter cells by micropinocytosis [140]. This mechanism is shared with α-synuclein but not with huntingtin, fibrils, possibly because both tau and α-synuclein contain heparin/heparan sulfate-binding domains which are required for HSPG binding [203]. In addition, Bin1, which increases the risk of developing late-onset AD and modulates tau pathology, affects tau propagation by negatively influencing endocytic flux [70, 246]. Thus, depletion of neuronal Bin1 enhances the accumulation of tau aggregates in endosomes [59]. Conversely, blocking endocytosis by inhibiting dynamin reduces the propagation of tau pathology [521].

Certain structural changes in tau, such as fragmentation and/or oligomerisation, appear to enhance the ability of tau both to aggregate and to propagate between cells. C-terminally truncated tau is abundant in synaptic terminals in aged control and AD brain [459]. Notably, depolarisation significantly potentiates tau release in AD nerve terminals compared to aged controls, indicating that tau cleavage may facilitate tau secretion and propagation from the presynaptic compartment [459]. When expressed in SH-SY5Y cells, the Tau_243–441_ (Tau-CTF24) fragment showed a higher propensity for aggregation than full-length tau, following exposure to extracellular insoluble tau seeds [327]. Tau_243–441_ inclusions from SH-SY5Y cell lysates also propagated more efficiently than inclusions generated from full-length tau [327]. Furthermore, Tau_243–441_ aggregates bound to cells more rapidly and in greater amount than aggregated full-length tau [327]. These results suggest that truncation of tau enhances its prion-like propagation and likely contributes to neurodegeneration.

Small tau oligomers have been suggested to be the major tau species undergoing tau propagation. Whereas oligomeric tau and short filaments of recombinant tau are taken up by primary neurons, tau monomers and long tau filaments purified from rTg4510 mouse brain are excluded [521]. Tau dimers and trimers isolated from PSP brain have also been shown to seed aggregation of 3R and 4R tau [157]. Notably, tau trimers also represent the minimal particle size that can be taken up and used as a conformational template for intracellular tau aggregation in human tau-expressing HEK293 cells [342]. In contrast, identification of the seeding-competent tau species in P301S tau transgenic mice revealed the requirement for large tau aggregates (>10 mers) [228]. However, there appear to be biochemical differences between aggregates formed from recombinant tau and inclusions isolated from P301S tau mice. Thus, recombinant tau aggregates are more resistant to disaggregation by guanidine hydrochloride and digestion by proteinase K, and display a lower seeding potency than those from P301S tau mice [350, 434]. These studies highlight the fact that the seeding competency of tau aggregates is dependent on both their size and conformation. It is clear that a balance between transmissibility and propensity to aggregate is required for effective inter-neuronal propagation of pathogenic tau species and resultant neurodegeneration [432].

An interesting aspect of the transmissibility of prions is the fact that different strains of prions induce distinct neurodegenerative phenotypes with reproducible patterns of neuropathology [245]. Tau exhibits a similar behaviour when brain homogenates prepared from different types of tauopathy, including AD, PSP and CBD, are injected into the brains of transgenic ALZ17 mice which overexpress 2N4R human tau [245]. The tau inclusions formed in the brain of injected ALZ17 mice closely resemble those in the originating source of brain extract. These findings support the view that during propagation tau forms multiple, stably propagating conformers with significant conformational and structural diversity. Fibrils formed of wild-type tau and mutant P301L/V337 M tau each have distinct secondary structures and morphologies [141]. Furthermore, wild-type tau aggregates induced by P301L/V337M tau seeds more closely resemble P301L/V337M fibrils than wild-type tau fibrils, and these are stable after repeated seeding [141]. Therefore, it appears that the nature of the tau seed is the determinant of the structural characteristics of the resultant tau aggregates [141]. This notion is further supported by the identification of two tau strains that induced distinct pathologies in cells and in vivo, each of which was passed on to daughter cells with remarkable fidelity [432]. In parallel, tau aggregates purified from AD or CBD brain also induce characteristic patterns of tau pathology in P301S tau mice [41]. The human tauopathy-derived aggregates affect different neural cell populations and distinct brain regions in the tau mice, suggesting that there may be additional parameters that discriminate between the tauopathies in different tau prion strains. In a recent study, 18 tau strains isolated from recombinant tau fibrils, mouse brain, and human brain, were found to display differing intracellular morphologies [245]. When injected into PS19 mice overexpressing human 1N4R P301S tau, these tau strains displayed differing spreading rates and preferences for specific brain regions, leading to a varied range of neuronal and astrocytic pathologies [245]. These results further reinforce the idea that differences between tau strains account for the diverse biochemical and phenotypic manifestations of tauopathies.

Tau propagation can be further accelerated through activation of inflammatory pathways. Exposure of extracellular misfolded truncated tau activates microglia through the MAPK pathway and induces the production of pro-inflammatory cytokines, including Interleukin-1β, Interleukin-6 and tumour necrosis factor alpha [263], which promote tau phosphorylation inside neurons [543]. Moreover, activated microglia may directly mediate tau propagation. The detection of tau in microglial-derived exosomes has demonstrated the involvement of microglia in tau propagation [22]. Around the same time, microglial activation resulting from deficiency of microglia-specific fractalkine receptor is found be preceding the spreading of tau pathology between anatomically connected hippocampal regions. Taken together, these results suggest that neuroinflammation could induce, or at least facilitate the propagation of tau pathology. Conversely, evidence showing microglial degeneration caused by soluble phospho-tau from AD hippocampi has also been reported [431]. Although the exposure of extracellular tau seemed to result in two conflicting outcomes, these findings are not naturally exclusive as the soluble fraction from AD hippocampi is consist of a mixture of tau species, which process different infectivity and cytotoxicity. It is also notable that the impact of neuroinflammation on tangle formation are rather limited and vice versa; no toxic effects of sarkosyl-insoluble tau on microglia was observed, providing peripheral evidence for the notion that more dynamic tau species are in the central stage during tau propagation [431, 543].

In summary, there is a significant body of evidence in support of the prion-like hypothesis for tau propagation, potentially through *trans*-synaptic transmission of pathological tau oligomers and/or aggregates [103, 302]. This could explain how the occurrence of an initial nidus of aggregated pathological tau may seed tau aggregation and then spread to more distal brain regions during the pathogenesis of tauopathy [162]. Meanwhile, a role of neuroinflammation in tau propagation has also been suggested shedding new light on potential therapeutic strategies. Furthermore, the discovery of both soluble and oligomeric forms of extracellular tau offers a plausible explanation for the apparent efficacy of tau antibody immunisation to slow disease progression in mouse models of tauopathy [23, 37, 44, 69].

## Tau targeted treatments

A variety of different therapeutic strategies have been examined for their efficacy in the tauopathies (reviewed in [172]). These approaches include reducing tau aggregation and/or preventing tau oligomer formation (reviewed in [52]). Small molecules that reduce tau aggregation in cell models and in transgenic mice, including methylthioninium derivatives, and other compounds identified through chemical microarray and library screens, have yet to be shown to be effective in human disease. For example, a Phase III clinical trial of leuco-methylthioninium bis(hydromethanesulfonate) (LMTM) has not shown benefit as an add-on treatment for mild-to-moderate AD [155]. However, it is possible that LMTM may yet prove to have some potential as a monotherapeutic agent and longer term clinical studies remain in progress to determine the efficacy of this tau disaggregating compound.

Previous clinical trials have targeted tau phosphorylation in AD and PSP by investigating inhibitors of the protein kinase GSK3. However, despite some of these compounds effectively reducing phosphorylated tau in CSF, none have shown efficacy in tauopathy [187]. A complementary approach is using MK-8719 (Alectos Therapeutics Inc, and Merck Sharpe and Dohme), an orally available small molecule, which selectively inhibits *O*-GlcNAcase, thereby potentially preventing phosphorylation at the same sites on tau. MK-8719 is designated as an orphan drug and a Phase I clinical trial for PSP has recently been completed, the outcome of which is awaited.

Alternative strategies to target tau include active immunisation with tau polypeptides, or passive immunisation using antibodies recognising tau. Testing of active tau immunisation in Phase II clinical trials for mild-moderate AD (NCT02579252) is ongoing for AADvac1 (Axon Neuroscience SE), which comprises misfolded tau, residues 294–305 (KDNIKHVPGGGS). An early obstacle to the implementation of tau antibody therapy was the supposition that antibodies would need to be internalised by neurons to be effective, and that immune modulation and microglial activation might also be problematic. Recently, however, it has become apparent that binding of antibodies to tau may be sufficient to alleviate the spread of tau pathology and potentially also disease pathogenesis [280]. Clinical trials of passive tau immunotherapy include the potentially therapeutic antibodies (1) ACI-35 (AC Immune SA and Janssen), which targets tau residues 393–408, phosphorylated at Ser396/Ser404 (VYKpSPVVSGDTpSPRHL), in a recently completed Phase Ib trial for mild-to-moderate AD, (2) C2N-8E12 (AbbVie and C2N Diagnostics), a humanised antibody that recognises residues 25–30 (DQGGYT) in aggregated tau, in Phase II trials for AD (NCT02880956) and PSP (NCT02985879), (3) BMS-986168 (Bristol-Myers Squibb), a humanised antibody targeting residues 9–18 (EVMEDHAGTY) of extracellular N-terminally truncated tau, about to enter a Phase II trial for PSP (NCT03068468), and (4) RO7105705 (AC Immune SA, Genentech, and Hoffmann-La Roche) an antibody targeting phosphorylated Ser409 in tau, in a Phase I trial for mild-to-moderate AD (NCT02820896). It is not yet clear which form, or which precise sequence of tau will be the most suitable target for immunotherapy, however, this may become apparent once the results from current clinical trials are reported. If a safe and effective tau-based vaccine can be produced, this would offer the possibility of providing long-term protection against the development of tauopathy.

## Concluding remarks

It is becoming clear that tau can undertake a multitude of roles beyond its most well established function of stabilising axonal microtubules. Functions now ascribed to tau include maintaining structural integrity, axonal transport, and signalling within and between neurons. These roles are facilitated by the finding that tau is located not only in axons but is also found in multiple neuronal compartments and in the extracellular spaces.

An intriguing and still poorly understood aspect of tau biology is the rationale for the existence of six alternatively spliced tau isoforms in the adult human CNS. The balance of tau isoforms in human brain is clearly important, since disrupted tau splicing with a consequent alteration in the ratio of tau protein isoforms is apparent in several tauopathies. Changes in the 4R/3R tau isoform balance are directly linked to many of the known causal mutations in *MAPT*. However, the fact that the tau isoform ratio is also affected in sporadic disease, in which no mutations in tau have been detected shows that tau splicing is regulated by factors other than *MAPT* transcript expression. This suggests the possibility of tau isoform-dependent degradation, which could be regulated by differential association of distinct tau isoforms with specific subcellular compartments or organelles. Maintaining a physiological balance of 4R/3R tau isoforms clearly has important implications for the tauopathies, since this affects maintenance of the microtubule cytoskeleton as well as having a potential impact on the association of tau with binding proteins and possible tau mislocalisation. It is important therefore to understand the biological importance of the expression of multiple spliced tau isoforms as well as the functions of each of the individual tau species. This knowledge may ultimately lead to identification of novel mechanisms involved in the development of tau pathology and disease pathogenesis in the tauopathies.

Another pressing area in the field is to better understand the functions of extracellular tau and the consequences of both physiological tau release and pathological tau propagation. The extrusion of soluble tau from neurons is a normal physiological event that is stimulated by neuronal activity. Furthermore, experiments in cell models have shown that tau aggregates can be taken up by and released from cells expressing aggregation-prone tau. The secretion and uptake of these different forms of soluble and insoluble tau most likely occur through distinct mechanisms. It is conceivable therefore that physiological secretion and uptake of soluble tau may initiate a potentially receptor-mediated component of intercellular signalling between neurons. In contrast, the release of aggregated tau is of particular interest in the tauopathies, since this could represent an attempt to remove potentially pathogenic tau from affected neurons. Such extracellular tau aggregates can be taken up by connected neurons, thereby providing a template for further misfolding of tau in unaffected neurons and resulting in the spreading of tau pathology. Discovering the temporal course of tau release and propagation during tauopathy development, and elucidating the molecular mechanisms underlying the release and uptake of both physiological and pathological forms of tau, are therefore important research goals.

There is increasing research interest in the involvement of tau in neurodegenerative disease, and the means by which new therapies can ameliorate tau-associated neurodegeneration. Therapeutic strategies aimed at reducing tau aggregation have not yet delivered the promise predicted from the results of experimental animal and cellular models. Moreover, this approach is complicated by the complex issue of whether highly aggregated forms of tau are toxic or protective to neurons. Thus, if tau tangles do provide some degree of neuroprotection, then disaggregating treatments could potentially exacerbate tauopathy through generating toxic, lower-order tau oligomers and soluble forms of phosphorylated tau. However, there is hope that clinical trials of tau immunotherapies may yet prove to be successful. One of the most pressing issues with tau-directed antibody approaches is to identify the precise form of tau, including either a specific region of the protein, a post-translational modification, or a distinct tau conformation that might be most efficiently targeted using this approach. Nevertheless, even in the absence of this knowledge, it seems feasible that either passive or active immunisation which results in an overall reduction in the total amount of tau could prove effective in tauopathies.
